# Increased Functional Stability and Homogeneity of Viral Envelope Spikes through Directed Evolution

**DOI:** 10.1371/journal.ppat.1003184

**Published:** 2013-02-28

**Authors:** Daniel P. Leaman, Michael B. Zwick

**Affiliations:** Department of Immunology and Microbial Science, The Scripps Research Institute, La Jolla, California, United States of America; University of Zurich, Switzerland

## Abstract

The functional HIV-1 envelope glycoprotein (Env) trimer, the target of anti-HIV-1 neutralizing antibodies (Abs), is innately labile and coexists with non-native forms of Env. This lability and heterogeneity in Env has been associated with its tendency to elicit non-neutralizing Abs. Here, we use directed evolution to overcome instability and heterogeneity of a primary Env spike. HIV-1 virions were subjected to iterative cycles of destabilization followed by replication to select for Envs with enhanced stability. Two separate pools of stable Env variants with distinct sequence changes were selected using this method. Clones isolated from these viral pools could withstand heat, denaturants and other destabilizing conditions. Seven mutations in Env were associated with increased trimer stability, primarily in the heptad repeat regions of gp41, but also in V1 of gp120. Combining the seven mutations generated a variant Env with superior homogeneity and stability. This variant spike moreover showed resistance to proteolysis and to dissociation by detergent. Heterogeneity within the functional population of hyper-stable Envs was also reduced, as evidenced by a relative decrease in a proportion of virus that is resistant to the neutralizing Ab, PG9. The latter result may reflect a change in glycans on the stabilized Envs. The stabilizing mutations also increased the proportion of secreted gp140 existing in a trimeric conformation. Finally, several Env-stabilizing substitutions could stabilize Env spikes from HIV-1 clades A, B and C. Spike stabilizing mutations may be useful in the development of Env immunogens that stably retain native, trimeric structure.

## Author Summary

A vaccine is needed to prevent HIV/AIDS but eliciting potent neutralizing antibodies (Abs) against primary isolates has been a major stumbling block. The target of HIV-1 neutralizing antibodies is the native envelope glycoprotein (Env) trimer that is displayed on the surface of the virus. Virion associated Env typically elicits antibodies that cannot neutralize primary viruses. However, because native Env trimers can dissociate and coexist with non-fusogenic forms of Env interpreting these results are difficult. Here, we used directed evolution to select for virions that display native Env with increased stability and homogeneity. HIV-1 virions were subjected to increasingly harsh treatments that destabilize Env trimers, and the variants that survived each treatment were expanded. We could identify seven different mutations in Env that increased its stability of function in the face of multiple destabilizing treatments. When these mutations were combined, the resulting mutant Env trimers were far more stable than the original Env protein. Incorporating trimer-stabilizing mutations into Env-based immunogens should facilitate vaccine research by mitigating the confounding effects of non-native byproducts of Env decay. A similar approach may be used on other pathogens with potential vaccine targets that are difficult to isolate and maintain in a native form.

## Introduction

For an HIV/AIDS vaccine to be effective, it is widely thought that it should elicit high titers of broadly neutralizing antibody (Ab) [1], [2]. HIV-1 neutralizing Abs target the envelope glycoprotein (Env) spike, which is a trimer containing three copies each of the surface subunit, gp120, and the transmembrane subunit, gp41 [3]. A major confounding issue in the rational development of Env as a vaccine is that fusion-competent Env trimers are often labile and heterogeneous, so distinguishing fusogenic from other forms of Env can be challenging [4]–[8]. Non-native forms of Env include dissociated gp120 monomers and dimers, gp41 stumps, monomers and oligomers of unprocessed gp160, as well as Env with aberrant disulfides and heterogeneous glycosylation [6], [7], [9]–[11]. In particular, non-native forms of Env may serve as immune decoys and elicit non-neutralizing Abs [6], [12]–[14]. Envs that are truncated prior to the gp41 transmembrane (TM) domain have in some cases been engineered as trimers, but these are not in a native conformation as, unlike native Env, they are typically recognized by non-neutralizing Abs and also elicit non-neutralizing Abs after immunization [15]–[20]. Thus, limiting exposure to the immune system of non-fusogenic forms of Env through stabilization of the native structure may facilitate HIV-1 vaccine design.

HIV-1 Env spikes are held together by non-covalent interactions among its subunits. Mutations that accelerate spontaneous or CD4 receptor-induced dissociation of gp120 from the HIV-1 Env complex can be found in various regions including the N-heptad repeat (NHR) [21], the disulfide loop (DSL) [22] and C-heptad repeat (CHR) regions [21], [23] of gp41, as well as in the C1 [24], V3 [25], β3–β5 loop of C2 [26], and C5 [27] regions of gp120. This may be expected on chance, as random mutations are much more likely to disrupt than stabilize the structure-function of a protein. Indeed, mutations that would stabilize Env trimers in the active membrane-anchored form have not been forthcoming or even reportedly sought after. One potential solution has been the introduction of a disulfide-bond between gp120 C5 and the DSL of gp41 (*e.g.* 501C and 605C; known as “SOS”), which, when exposed to a reducing agent, breaks and allows for productive entry of SOS-modified HIV-1 into target cells [28], [29]. However, the disulfide bond is subject to exchange and can also leave many non-neutralizing epitopes exposed, at least in soluble forms of the SOS molecule [30], [31]. Thus, we envisioned an alternative strategy that allows the virus to select for mutations that stabilize the Env trimer naturally, without compromising native structure or antigenicity.

We have shown previously that, depending on the viral isolate, virion associated Env can have different levels of heterogeneity and can have a range in stabilities to conditions such as elevated temperature, prolonged incubation at 37°C or exposure to denaturants [6], [7]. Env from the clade B isolate ADA is both labile and heterogeneous [7]. An ADA variant, AD8, has recently been associated with rapid elicitation of broadly neutralizing Abs in a macaque model and so is relevant to vaccine research [32]. Heterogeneous and labile Envs can be problematic to study because non-fusogenic forms of Env can accumulate and confound measurements [7]. Diversity in glycosylation may account for some of the observed heterogeneity, even in functional Env, as certain cloned isolates of HIV-1 are incompletely neutralized by glycan-sensitive Abs such as PG9 and PG16 [10].

Directed evolution is often used in virology to study cellular tropism or resistance to neutralizing antibodies or antiviral drugs. HIV-1 has been selected to resist spontaneous inactivation of Env at cold temperatures or to overcome functional defects associated with truncated Envs [33], [34]. Increasing the stability and homogeneity of native Env might also improve Env-based vaccines by limiting potentially distracting non-neutralizing and immunogenic surfaces of Env, improve correlations between observed Env structures and their associated functions, as well as inform the design of more molecularly defined immunogens [4]–[6]. We therefore devised a strategy in which Env is selected specifically for increased stability in its unliganded, functional state. Thus, HIV-1 is subjected to iterative cycles of harsh destabilizing conditions and subsequent viral expansion. We show that this approach can overcome as well as help understand the molecular heterogeneity and lability of viral spikes, which may have implications for the design of immunogens based on functional, unliganded and membrane-anchored Env of multiple clades of HIV-1.

## Results

### Production of HIV-1 mutant pools and their selection using destabilizing conditions

We previously showed that Env from the clade B, R5 isolate ADA was relatively heterogeneous as well as labile to heat, guanidinium hydrochloride (GuHCl) and to spontaneous inactivation at physiological temperature [7]. We generated mutant pools of HIV-1 ADA using site-directed mutagenesis targeted to regions of Env shown previously to affect Env trimer stability (**Figure S1A**). However, random mutant clones were found to be mostly non-infectious and when library DNAs were transfected into 293T cells, the virus produced was of very low titer, most likely because of the high number of mutations targeted to conserved regions of Env (**Figure S1B and C**). Nevertheless, extensive passaging in MT2-CCR5ΔCT cells yielded high titer virus with each pool (**Figure S1D**).

**Figure 1:**
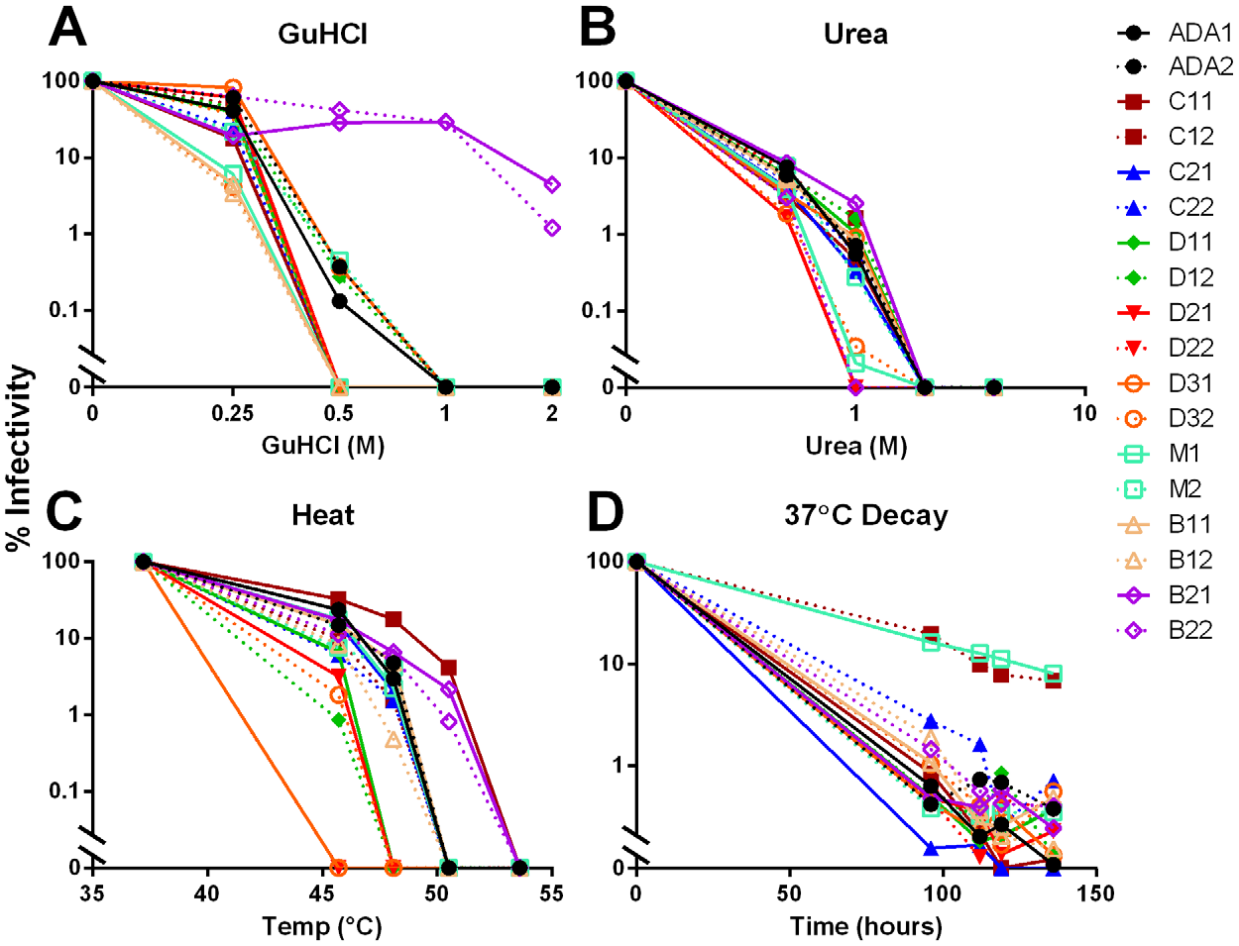
Virion pools are resistant to destabilizing conditions after three rounds of selection. Virion pools that had already been selected for two rounds in duplicate were treated with either GuHCl (**A**), urea (**B**), or heat (**C**) for one hour, or incubated at 37°C for up to 144 hours (**D**). After treatment, an aliquot of virus was removed and titered on TZM-bl cells. Percent infectivity remaining was calculated relative to untreated virus.

To select for stable ADA variants, the pools of virions were treated separately with incremental concentrations of GuHCl, urea, hyper-physiological temperatures or prolonged incubations at physiological temperature that inactivates wild-type ADA [7]. After destabilizing treatment, the surviving infectious viruses were rescued on MT2-CCR5ΔCT cells. Following three rounds of selection, some virion pools were more stable than ADA wild-type to GuHCl and heat treatment (e.g. B21 and B22), one was resistant to heat (e.g. C11), and two pools were resistant to 37°C decay (e.g. C12 and M2; **Figure 1**). None of the selected library pools were found to have increased stability to urea.

### Identifying Env clones from stability-enriched virus pools

**Figure 2:**
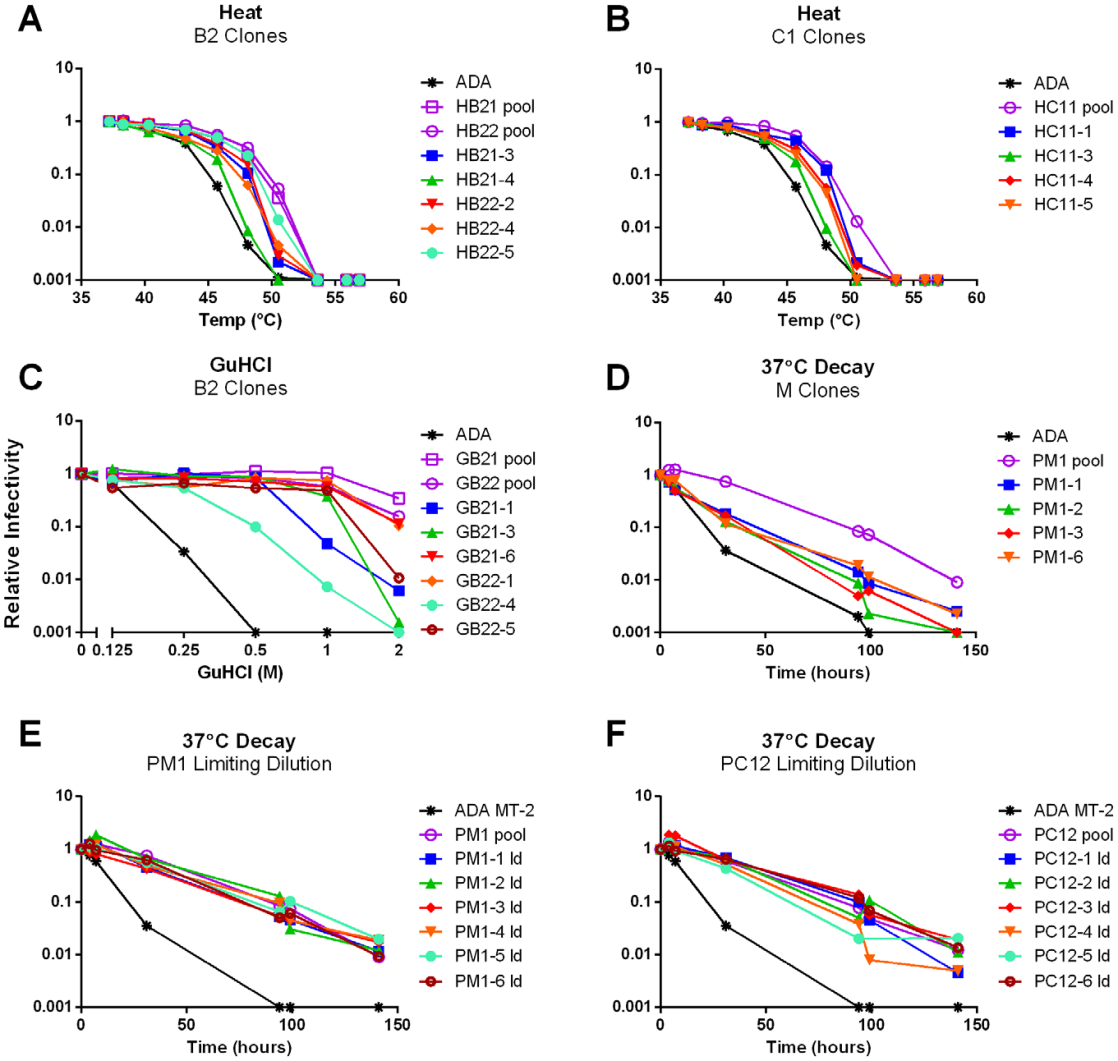
Identification of stable clones from virion pools. (**A–D**) Stable Env cDNAs were rescued using RT-PCR on pooled viral RNA. Variant Env clones derived from the heat-selected B2 (**A**; HB2) and C11 (**B**; HC11) virion pools, the GuHCl-selected B2 pools (**C**; GB2), and the 37°C-selected M1 pool (**D**; PM1) were tested for resistance to the destabilizing treatment with which they were selected alongside the originating virion pool. (**E** and **F**) Stable HIV-1 variants identified via limiting dilution. Polyclonal pools of virus derived from the 37°C-selected M1 (**E**; PM1) and C12 (**F**; PC12) virion pools were subjected to limiting dilution and individual clonal/oligoclonal viruses were tested for infectivity decay at 37°C.

To identify individual Env mutants of increased stability, viral RNA was purified from the stability-enriched pools and *env* was amplified using RT-PCR. Only the ectodomain portion of Env was subcloned back into the pLAI display vector in order to rule out mutations in the gp41 TM and cytoplasmic tail (CT) domains that might affect interactions below the viral membrane, such as between the gp41 CT and Gag [35], and would add further complexity to the analysis. Individual Env clones were picked from each pool and the corresponding virions were assayed for resistance to each of the selection conditions (**Figure 2**). A variety of stability phenotypes were observed. Notably, some clones from the GuHCl and heat-treated pools fully recapitulated the stability phenotype of the originating viral populations (**Figure 2A, B, and C**).

For virion pools that showed resistance to decay at 37°C (i.e. PM and PC1), none of the cloned Envs approached the stability of the corresponding pool (**Figure 2D** and data not shown). We therefore took an alternative limiting dilution approach to identify stable mutants. Using MT2-CCR5ΔCT cells as target cells, six limiting dilution wells for each pool were found to be equal in stability to the parental pools (**Figure 2E and F**). In summary, we successfully identified HIV-1 mutant clones that were stable to the same conditions as the pools from which they were derived.

**Figure 3:**
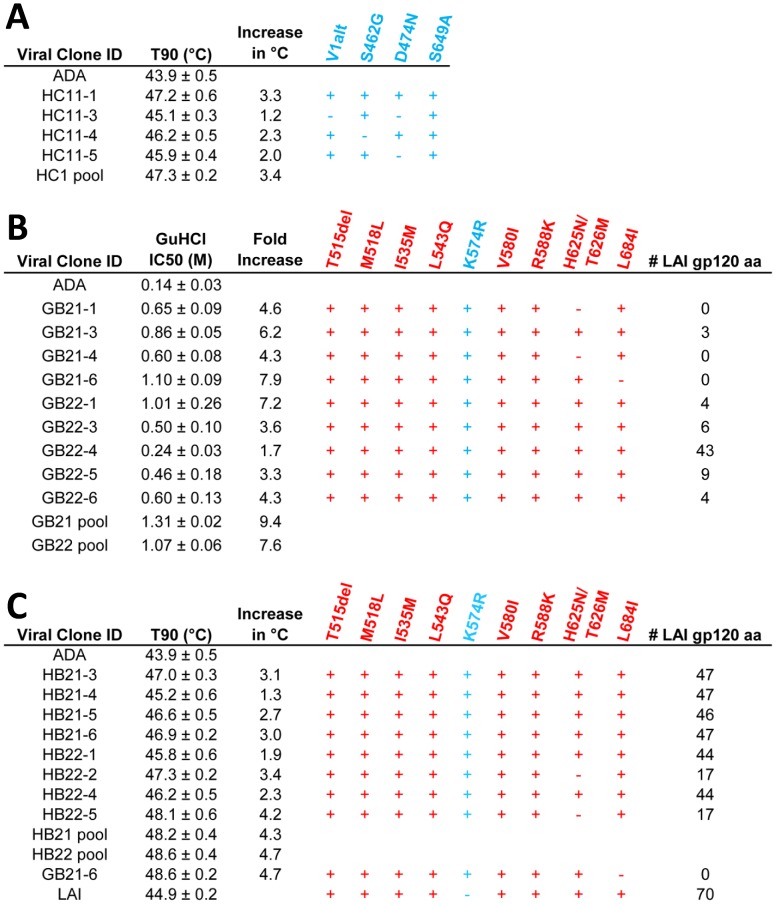
Identification of mutations responsible for the increased functional stability of Env. Individual Envs from (**A**) HC11, (**B**) GB2, and (**C**) HB2 stability-selected pools were sequenced. For each variant, the relevant numerical measurement of stability (T90 (*i.e.* the temperature at which viral infectivity is diminished by 90% in one hour) for heat and IC50 for GuHCl+/− SEM), the corresponding increase in stability relative to wild-type ADA, and the presence (+) or absence (−) of a given mutation is indicated. Mutations that arose *de novo* are shown in blue and mutations derived from the HIV-1 isolate LAI are indicated in red. Shown also in (**B**) and (**C**) are the numbers of residues in gp120 of each variant that originated from LAI rather than ADA and that differ from ADA. V1alt in (**A**) refers to the combinatorial deletion of amino acids N139 and I140 and the substitution N142S.

To understand the basis of the hyperstable phenotypes of the rescued mutant clones, we determined the primary Env sequences. Mutations were identified, remarkably, only in regions of ADA Env not targeted by our mutagenesis procedure, most likely because the targeted mutations negatively impacted infectivity and any mutants were out-competed by the small number of virions incorporating mostly wild-type Env sequence. From the heat-stable library pool HC11, all of the clones contained the substitution S649A in the CHR region of gp41, whereas some but not all clones contained two substitutions in gp120 C5 (S462G and D474N) and an alteration to gp120 V1 (*i.e.* deletion of N139/I140 plus an N142S substitution that will hereby be referred to as “V1alt”). These mutations in the HC11 clones appeared to arise *de novo*. (**Figure 3A**).

**Figure 4:**
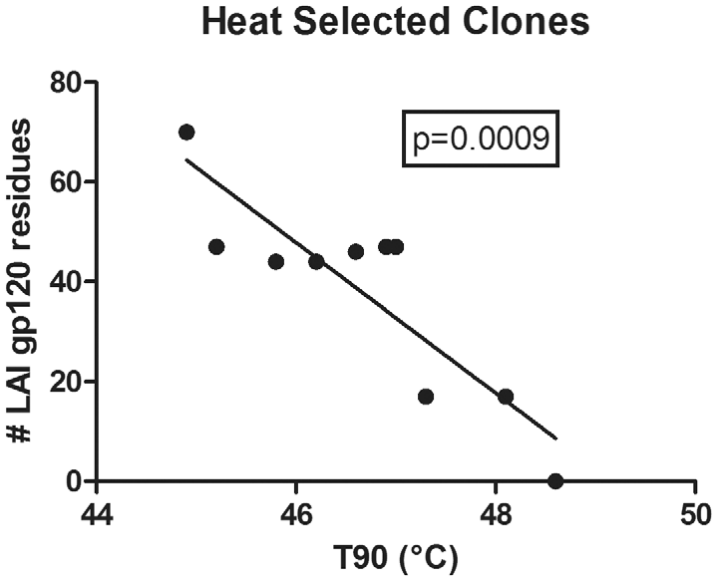
Env stability is inversely correlated with LAI-derived gp120 amino acid residues in HIV-1 variants. The T90 of each clone is plotted against the number of gp120 residues in each clone derived from LAI rather than ADA. Data for GB21-6, which contains LAI gp41 and ADA gp120, and that of LAI are also plotted and are the most and least stable viruses, respectively. The inverse correlation is statistically significant (p = 0.0009, F test).

Sequencing of Env from the GuHCl-stable and heat-stable library pools, G and HB2, surprisingly revealed mutations to residues in common with the LAI strain that was used in engineering the display vector, pLAI (**Figure 3B and 3C**). In these clones, recombination events involving a very small amount of LAI DNA appear to have occurred during the PCR used in library production. Importantly, all of the selected clones were more stable than either the parental strain, ADA, or the LAI strain (**Figure 3C**). Multiple recombination events appear to have taken place during the selection process resulting in virions containing different amounts of LAI-derived gp120 sequence, and among the most stable clones, LAI-derived sequence from gp41, but not from gp120, was associated with improved Env stability. Thus, the presence of LAI gp120 was significantly inversely correlated with Env stability (**Figure 4** **)** and the most stable clone (GB21-6) contained full LAI gp41 and ADA gp120. In total, 9 amino acid residues in the gp41 ectodomain differ between ADA and LAI (**Figure 3**). In addition, a tenth mutation, K574R, arose *de novo* and was conserved amongst the stable clones.

### Functional stability of mutant Envs extends to multiple destabilizing conditions

**Figure 5:**
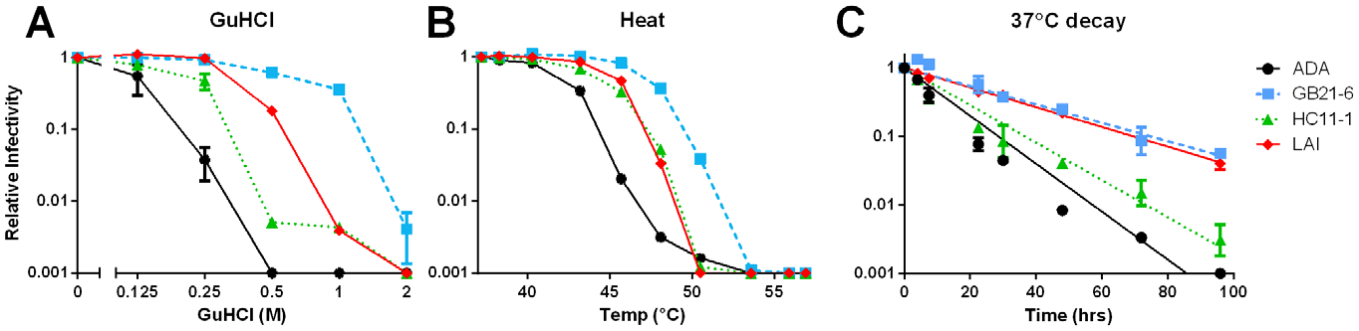
Stability selected ADA mutant HIV-1 are resistant to multiple destabilizing treatments. Two representative stable clones (GB21-6 and HC11-1) were tested along with ADA and LAI for resistance to GuHCl (**A**), heat (**B**), and 37°C decay (**C**). Shown are the results of a representative experiment performed in duplicate.

The stability of the selected Env clones can either be specific to the selection conditions, or can impart a broader resistance to multiple destabilizing treatments. To investigate, the most stable clones from the GuHCl and heat-selected pools, GB21-6 and HC11-1, were subjected in parallel to GuHCl, heat and prolonged incubation at physiological temperature. Both GB21-6 and HC11-1 maintained infectivity with increased resistance to each of these conditions relative to wild-type ADA (**Figure 5**). Clone GB21-6 was consistently the most stable in each case. In keeping with our prior observation of a negative correlation between the number of LAI gp120 residues and Env stability (**Figure 4**), LAI was less stable than GB21-6 when treated with heat or GuHCl, but notably the two viruses displayed similar rates of decay at physiological temperature (**Figure 5**).

### Native PAGE reveals enhanced oligomeric stability and homogeneity among selected Env mutants

**Figure 6:**
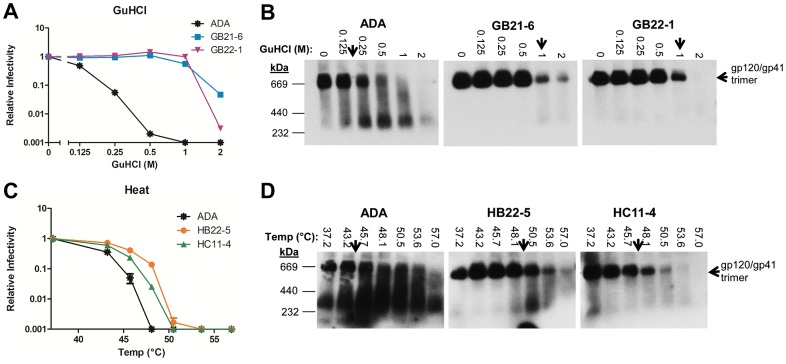
BN-PAGE and infectivity decay analysis reveals that functional stability of Env correlates with oligomeric stability. Two stable clones that were selected to be resistant to GuHCl (**A** and **B**) or heat (**C** and **D**) were exposed to the corresponding treatment. Afterwards, virus was assayed for infectivity in TZM-bl cells (**A** and **C**) and Env trimer dissociation was visualized by BN-PAGE Western blot using an anti-gp41 mAb cocktail (**B** and **D**).

To determine the relationship between functional stability and oligomeric stability of the unliganded mutant Env trimers, we turned to BN-PAGE. Two GuHCl resistant and two heat resistant clones were chosen for the analysis: GB21-6, GB22-1, HB22-5 and HC11-4, the latter of which shares similar stability and most of the same mutations as HC11-1 (**Figure 3**). The clones were subjected to increasing temperature or GuHCl concentrations, and samples were analyzed for infectivity and by BN-PAGE. In each case, dissociation of the Env trimer on BN-PAGE closely correlated with the loss of infectivity, with stability selected Envs clearly maintaining trimeric association under conditions that caused dissociation of wild-type ADA trimers (**Figure 6**). HC11-1, used above and in subsequent analyses, was also found to be more stable (data not shown). Importantly, the stable Env variants also appear more homogeneous than that of wild-type ADA as non-trimeric species in the former are much less apparent than in the latter.

### Stable Env clones are resistant to inactivation by Abs and other ligands

**Figure 7:**
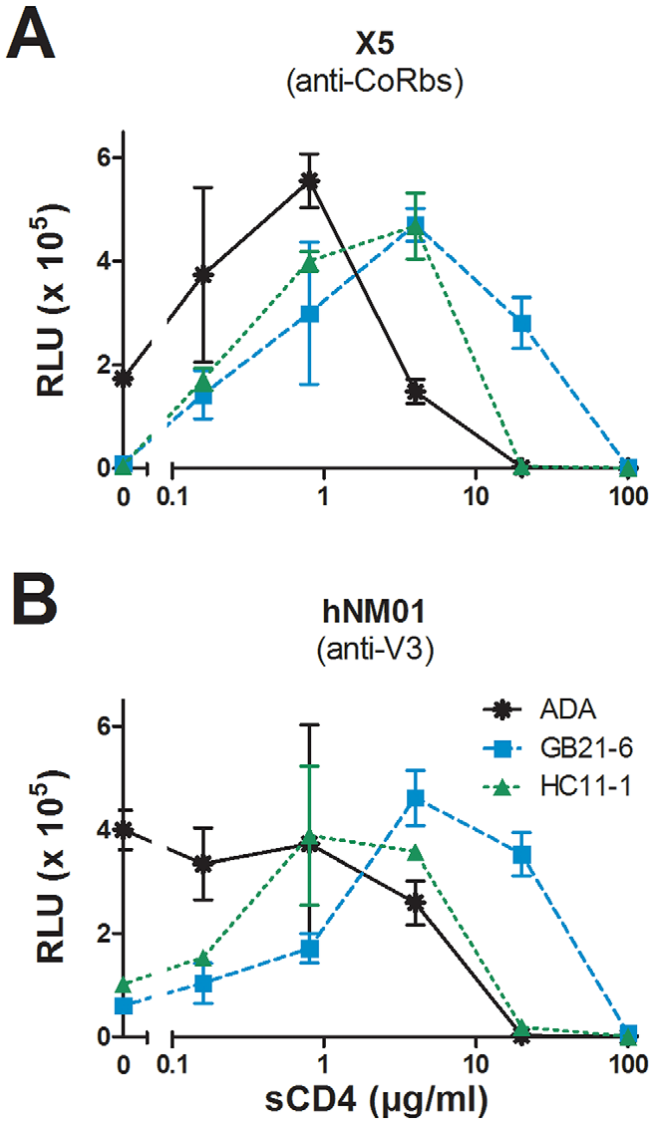
Virus capture assay shows that stable Env spikes are resistant to sCD4-induced inactivation. Wild-type ADA, GB21-6, and HC11-1 were pre-incubated with varying concentrations of sCD4 and then captured on microwells coated with either mAb X5 (**A**) or hNM01 (**B**). Unbound virus was washed away and the infectivity of the remaining virus was assessed using overlaid TZM-bl target cells. Experiments were performed in triplicate.

Certain neutralizing monoclonal Abs (mAbs) and other inhibitory ligands, such as soluble CD4 (sCD4), can destabilize and irreversibly inactivate the Env trimer upon binding [36]–[38]. We used two approaches to determine whether the stabilized clones of HIV-1 Env would resist ligand destabilization. In the first approach, we employed a modified virus capture assay (VCA) that was designed to crudely mimic Env destabilization through host receptor engagement. Thus, GB21-6 or HC11-1 virions were incubated in solution with increasing concentrations of sCD4 for 15 min, and then overlaid on microwells coated with either mAb hNM01 (anti-V3) or X5 (anti-coreceptor binding site (CoRbs)). Unbound virus was then washed away and TZM-bl target cells were overlaid to measure remaining infectivity. Binding of sCD4 to the Env trimer initially exposes the V3 loop and CoRbs in a fusion-active state [39], but after a short period the sCD4-bound trimer decays to an inactive state [36]. The VCA design provides an aggregate measure of induction of conformational changes by sCD4 and the functional stability of the sCD4-activated state. As expected, capture of infectious wild-type ADA was increased by low concentrations of sCD4 that promotes exposure of the epitopes of the capture mAbs, but decreased at higher concentrations as virions were inactivated (**Figure 7**). By contrast, when using the same initial concentrations of sCD4 capture efficiency of infectious virus was increased for both GB21-6 and HC11-1, and, at high concentrations of sCD4 that inactivated wild-type ADA, the infectivities of GB21-6 and HC11-1 were still intact. We note that in the absence of sCD4 mAbs X5 and hNM01 captured lower levels of GB21-6 and HC11-1 relative to wild-type ADA (**Figure 7**). This may be related to the BN-PAGE results showing that these viruses display more homogeneous trimers (**Figure 6**), as mAb X5 does not appear to bind the unliganded trimers of most primary isolates and likely captures virions via non-native Env [6].

**Figure 8:**
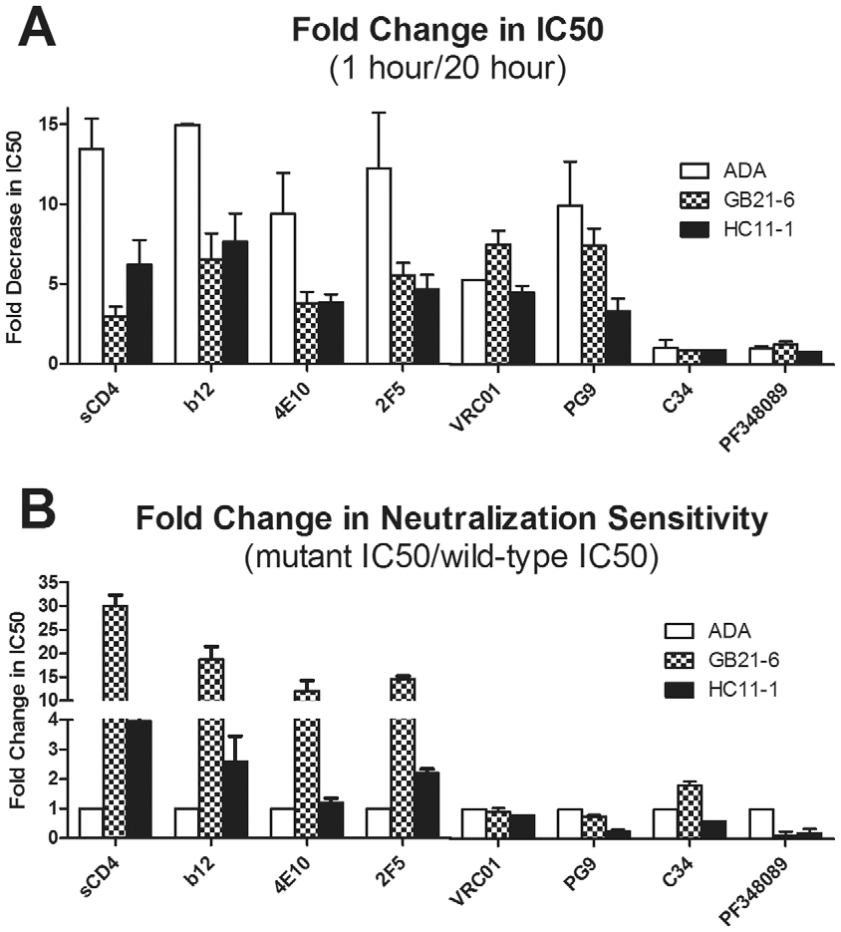
Stable HIV-1 Env mutants are resistant to ligand destabilization in infectivity assays. Wild type HIV-1 ADA and corresponding mutants were treated with the indicated neutralizing mAbs or inhibitors for either one hour or 20 hours, overlaid on TZM-bl target cells, and luciferase activity was measured 48 hours later. PF-348089 is an analogue of the inhibitor BMS-378806. IC50s were calculated from experiments performed in triplicate. (**A**) Plotted on the Y-axis for each ligand is the ratio of IC50 following a 20 hour pre-incubation period relative to a 1 hour pre-incubation period. (**B**) Plotted on the Y-axis is the ratio of IC50 of the stable Env mutant relative to wild-type ADA (mutant IC50/wild-type IC50) for each ligand using a 1 hour pre-incubation period.

If an inhibitor binds and inactivates Env, the IC50 of that inhibitor is expected to decrease over time [37]. In a second approach to assay ligand-induced Env destabilization, GB21-6 and HC11-1 viruses were pre-incubated with mAbs or inhibitors for two different periods of time prior to measuring viral infectivity on target cells. Of the inhibitors we tested, mAbs b12 (anti-CD4 binding site; CD4bs), 4E10 and 2F5 (anti-membrane proximal external region; MPER), as well as sCD4 have all been shown to destabilize Env trimers [36], [37]; mAb VRC01 (anti-CD4bs) has a much weaker destabilizing effect [40]; mAb PG9 (anti-V2/V3) has not been well studied in this context [41]; C34 (anti-NHR) should have no activity towards unliganded Env as it only binds Env post-CD4 engagement [42]; and PF-348089 is an analogue of BMS-378806 that binds to gp120 and prevents CD4-induced conformational changes [43], [44]. Consistent with the VCA results above, both GB21-6 and HC11-1 resisted inactivation by sCD4; similar resistance was also observed using b12, 4E10 and 2F5 (**Figure 8A** **; Table S1**). Thus, whereas the IC50s of these inhibitors against wild-type ADA decreased 9–15-fold from the first (1 h) to the second (20 h) pre-incubation time point, the IC50 decreases with GB21-6 and HC11-1 were only 3–6-fold and 4–8-fold, respectively. Notably, VRC01 affected all three viruses equivalently. PG9 inactivated both wild-type ADA and clone GB21-6 with IC50 decreases of ∼10-fold between pre-incubation times. In contrast, HC11-1 resisted PG9 inactivation, but this clone contains an alteration in V1 that may directly affect the PG9 epitope. Finally and as expected, C34 and PF-348089 did not inactivate any of the viruses over time.

The above inhibition data can also be expressed as a ratio of IC50s of the mutants to that of wild-type ADA using the standard 1 h pre-incubation time (**Figure 8B** and **Table S1**). Expressed in this way, GB21-6 is 12–30-fold more resistant than wild-type virus to sCD4, b12, 4E10 and 2F5, while HC11-1 is also resistant to these inhibitors, but to a lesser extent. The same two mutants are not generally more resistant to VRC01, PG9, and C34, with some exceptions that are most likely due to sequence changes that directly affect ligand binding (**Figure 3**). Hence, the stable clones tend to be more resistant to ligand-induced inactivation, except when the ligand is not of the destabilizing type (*e.g.* VRC01 and C34) or the inhibitor epitope is affected. Interestingly, both GB21-6 and HC11-1 were 5–10-fold more sensitive to PF-348089, an inhibitor that prevents CD4-induced conformational changes presumably by stabilizing the CD4-unbound state.

**Figure 9:**
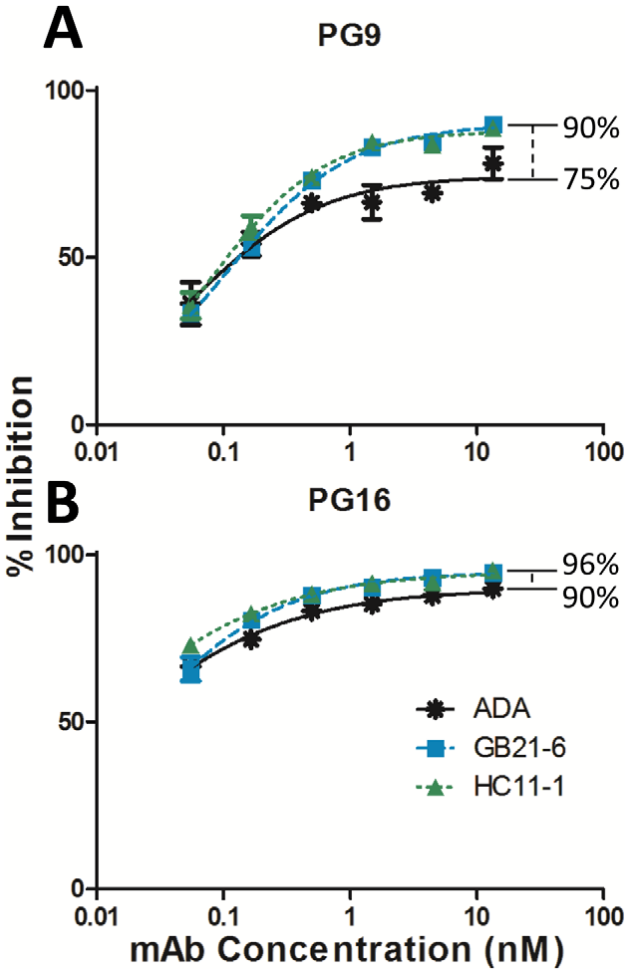
Stable HIV-1 Env mutants are more fully neutralized than wild-type ADA by mAbs PG9 and PG16. Viruses were pre-incubated with PG9 (**A**) or PG16 (**B**) for one hour, and then the mixture was overlaid onto TZM-bl target cells. Infectivity was measured 48 hours later. A representative experiment performed in triplicate is shown. Maximal percent inhibition (MPI) of the mAbs against each virus was calculated by non-linear regression of the neutralization curves.

It has been shown that mAbs PG9 and PG16 can neutralize ∼92% of HIV-1 isolates in a large multi-clade viral panel but can occasionally give rise to inhibition curves that plateau below 100% neutralization for certain sensitive isolates [10], [41]. The latter phenomenon is thought to be caused by heterogeneity in glycosylation on the Env trimer [10]. We observed such a phenomenon with wild-type ADA in which PG9 and PG16 exhibited a maximal percent inhibition (MPI) of 74% and 90%, respectively (**Figure 9**). Interestingly, both GB21-6 and HC11-1 exhibited higher MPIs against both mAbs; PG9 and PG16 had 90% and 96% MPI, respectively. Thus, not only do these two mutants of ADA exhibit decreased levels of heterogeneity and “cleaner” trimer bands on BN-PAGE, but they also show less heterogeneity within the pool of functional trimers as probed by mAbs PG9/16.

### Single amino acid substitutions in the gp41 NHR, CHR, and gp120 V1 regions stabilize functional Env

**Figure 10:**
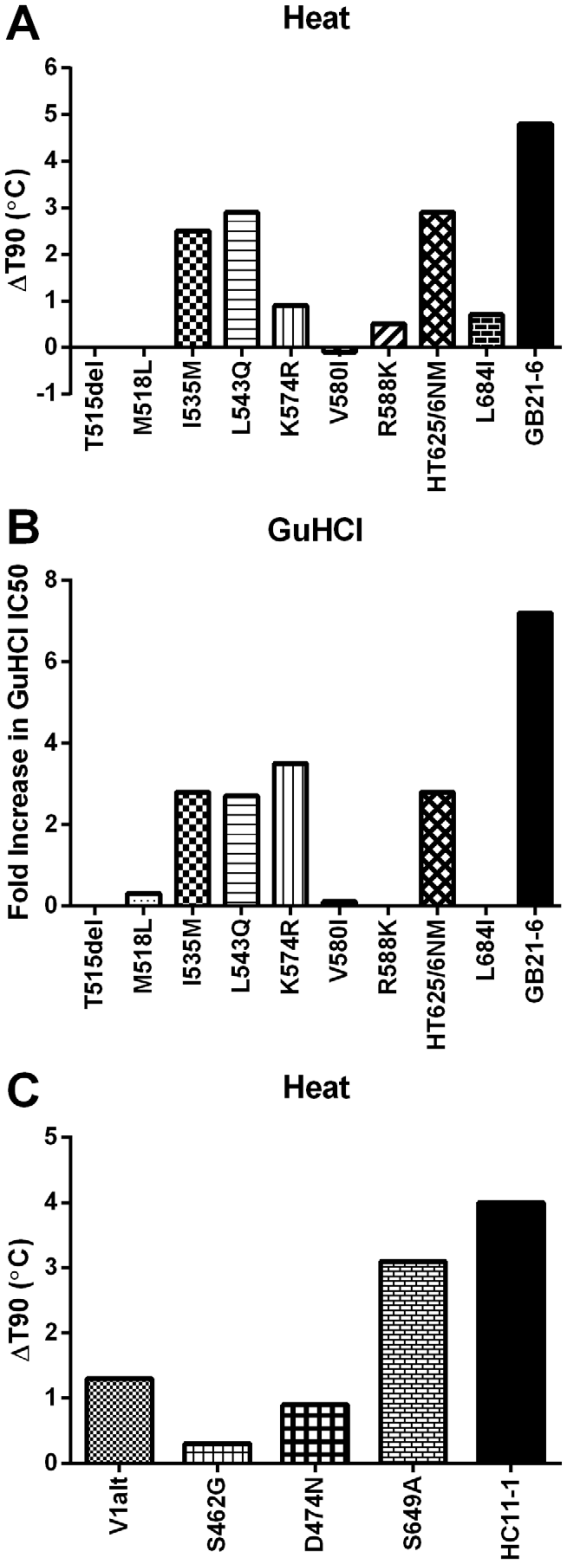
Effect of individual point mutations on stability of functional Env. Each of the sequence changes identified in the stable variants were introduced as point mutants into wild-type ADA. The mutations identified in the HB2 and GB2 variants were tested against heat (**A**) and GuHCl (**B**). The mutations derived from the HC11 variants were assayed only against heat (**C**). The change in T90 or in the IC50 for mutant relative to wild-type ADA is plotted for each point mutant.

Based on the stable mutant Env sequences we selected single amino acid residue changes to introduce into wild-type ADA and examined their effect on Env stability. H625N and T626M were introduced as a double substitution, as these residues were adjacent to one another and seemed to co-vary. Although none of the point mutants completely recapitulated the phenotype of the stable clones, stabilizing effects were clearly observed and could be narrowed down to a few residues in each case (**Figure 10**). Thus, from the B2 pools, I535M, L543Q, and K574R in the NHR and H625N/T626M in the CHR each partially stabilized ADA Env to both heat and GuHCl treatment. In the case of the HC11 clones, the CHR mutation S649A played the largest role in stabilization and the V1alt substitution provided a more limited increase in Env stability.

### Position 574 in gp41 strongly affects Env stability in both unliganded and liganded states

**Figure 11:**
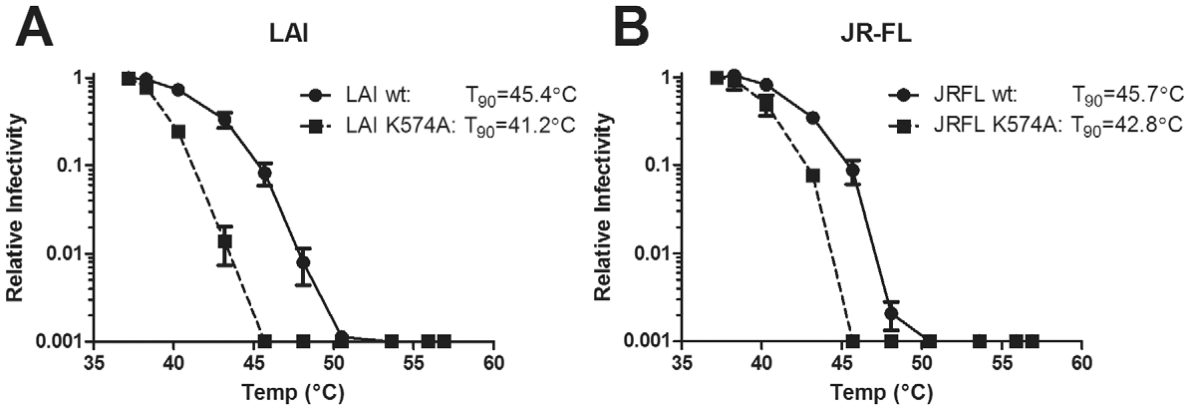
Substitution K574A in gp41 decreases the thermostability of HIV-1 functional Env. HIV-1 mutant K574A was tested for resistance to heat in either the LAI (**A**) or JR-FL (**B**) backgrounds relative to wild-type. Viruses were exposed to varying temperatures for one hour and assayed for infectivity using TZM-bl target cells. Results shown are the average of two experiments performed in duplicate.

Among the gp41 amino acid changes identified in the GB2 and HB2 virion pools that increased functional trimer stability, K574R was the only mutation that was not of LAI origin. The conserved Lys at position 574 residue has previously been shown to be crucial for stability of the six-helix bindle (6HB) protein that is a mimetic of gp41 in a post-fusion form [45]. Other studies have shown that mutations to the NHR of gp41 can affect neutralization sensitivity of HIV-1 [46], [47], and as shown above Env trimer stability can also alter sensitivity to certain inhibitors. To further characterize the relationship between Env stability and neutralization sensitivity due to mutation in the NHR, we examined how non-conservative mutation at position 574 affects trimer stability. We first performed this analysis using the mutant K574A in the LAI strain, which was generated in a previous study [48]. The K574A mutation profoundly decreased the T90 (the temperature at which viral infectivity is diminished by 90% in one hour) of HIV-1 LAI by ∼4°C and also globally increased sensitivity to a number of Env-destabilizing ligands (*e.g.* b12, sCD4, 2F5, 4E10, and b6; **Figure 11A** and **Table 1**). The most profound effect was observed with the weakly neutralizing CD4bs mAb b6 which was 250-fold more potent against K574A than wild-type LAI. In contrast, the substitution K574A had a less pronounced effect on neutralization by the non-destabilizing mAb 2G12 [37], and was only 2-fold more sensitive to mAbs and inhibitors that target Env in a pre-fusion intermediate state.

**Table 1:** Effect of substitution K574A in gp41 on sensitivity of HIV-1 to neutralizing mAbs and entry inhibitors.

	IC50 (nM) against LAI			IC50 (nM) against JRFL		
Inhibitor	Wild-type	K574A	IC50 Fold Decrease	Wild-type	K574A	IC50 Fold Decrease
b12	3.1	0.13	24	0.21	0.076	2.7
sCD4	5.4	0.26	21	12	3.9	3.0
2F5	12	0.13	91	6.0	0.84	7.2
4E10	13	0.27	48	19	2.2	8.8
b6	34	0.14	250	4200	85	49
2G12	2.9	0.72	4.0	2.2	1.1	2.1
T-20	1.8	0.87	2.1	3.3	0.93	3.5
8K8	540	260	2.1	*nd* *	*nd*	*nd*
PF-68742	590	290	2.1	*nd*	*nd*	*nd*

* nd: not determined.

We also introduced the K574A mutation into the relatively stable primary isolate, JR-FL. Again, K574A destabilized JR-FL (*i.e.* T90 decreased by ∼3°C) and also resulted in broader sensitivity to a variety of destabilizing inhibitors (**Figure 11B** **;** **Table 1**). In particular, K574A made JR-FL ∼50-fold more sensitive to b6. Overall, our results suggest that residue K574 also plays a crucial role in regulating stability of the receptor-naive Env trimer.

### ADA-stabilizing mutations can also stabilize Envs from other clades

To see if the stabilizing substitutions we identified could stabilize other viral Envs, we first targeted the relatively stable and homogeneously trimeric Env, JR-FL. L543Q, K574R, S649A, and V1alt were introduced, and, since JR-FL already contained M535 and N625/M626, the reverse mutations M535I and NM625/6HT were introduced to see if they would destabilize the trimer. When the mutants were tested for stability in the heat gradient assay, L543Q, K574R, and S649A all increased the T90 of JR-FL by 0.7–1.5°C, while M535I and NM625/6HT both decreased the T90 of JR-FL by ∼2°C (**Table 2**). V1alt did not have any effect on JR-FL stability, as might be expected due to the extreme sequence variability in this region. Thus, substitutions to these amino acid residues in the heterologous isolate JR-FL have similar effects on Env stability as in ADA.

**Table 2:** Effect of mutations on the functional thermostability (T90) of Env from heterologous primary isolates.

Virus	Mutant	T90 (°C)*	ΔT90 (°C)†
**JRFL C**lade B	wild-type	48.1±0.3	
	L543Q	49.3±0.4	+1.2
	K574R	49.6±0.4	+1.5
	S649A	48.8±0.4	+0.7
	M535I	47.3±0.2	−2.3
	NM625/6HT	46.3±0.2	−1.8
	V1alt	48.1±0.2	0.0
**RHPA4259** Clade B	wild-type	44.5±0.1	
	I535M	45.6±0.4	+1.1
	L543Q	45.7±0.1	+1.2
	K574R	45.8±0.5	+1.3
	S649A	45.3±0.3	+0.8
**Q769.b9** Clade A	wild-type	43.8±0.3	
	I535M	43.6±0.8	−0.2
	K574R	44.6±0.4	+0.8
	S649A	44.9±0.2	+1.1
**ZM109F** Clade C	wild-type	44.9±0.1	
	I535M	45.2±0.2	+0.3
	K574R	46.4±0.2	+1.5
	S649A	44.5±0.1	−0.4

* T90: the temperature at which viral infectivity is decreased by 90% in 1 hour.

† The change in the T90 of the Env mutant relative to the corresponding wild-type virus.

Next, we introduced stabilizing substitutions into isolates from multiple clades that have previously been shown to be labile, including Q769.b9 (clade A), RHPA4259 (clade B) and ZM109F (clade C) [7]. Substitutions I535M, K574R and S649A were introduced into all three strains; L543Q was only introduced into RHPA4259, as Q769.b9 and ZM109F already contained Q543; all three isolates already contained N625/M626. All mutations inserted into the clade B isolate RHPA4259 increased its T90 by 0.8–1.3°C (**Table 2**). Substitutions in the non-clade B isolates showed mixed effects. Thus, K574R increased the T90 of both isolates, S649A increased the T90 of Q769.b9 but not ZM109F, and I535M did not affect the stability of either strain. Thus, the stabilizing mutations identified in this study appear to have a similar effect on all clade B isolates tested, and some of the substitutions (*i.e.* K574R and S649A) impart a stabilizing phenotype on Envs across the three major clades of HIV-1.

### Substitutions from unrelated Env mutants combine to further improve Env trimer stability

**Figure 12:**
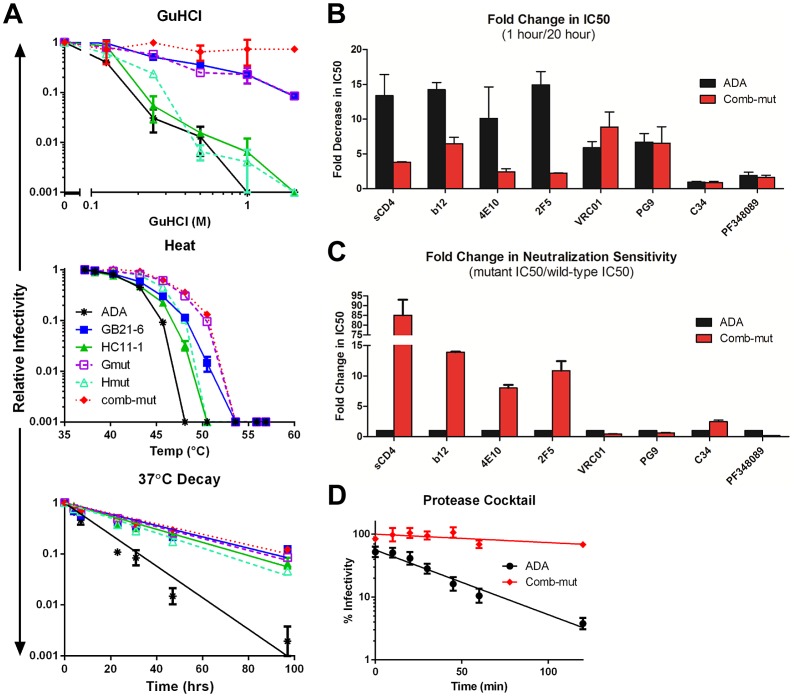
Combining stabilizing point mutations from different Env variants produces a hyperstable virus, comb-mut. Point mutations derived from GB21-6 or HC11-1 that were also found to increase stability of ADA (**Figure 10**) were combined into viruses, Gmut and Hmut, for comparison to the original clones, while all seven stabilizing mutations were combined to produce comb-mut. (**A**) These viruses with combined Env mutations were tested for resistance to GuHCl (top), heat (center), or 37°C decay (bottom). Results shown are from a representative experiment performed in duplicate. (**B**) The relative resistance of comb-mut to ligand-induced infectivity decay is shown as a function of the fold decrease in IC50 after 20 hour pre-incubation relative to 1 hour pre-incubation as described in **Figure 8**. (**C**) The relative sensitivity of comb-mut to neutralization by a panel of ligands is shown as the ratio of mutant IC50 to wild-type IC50 as described in **Figure 8**. (**D**) ADA and comb-mut virions were incubated with a cocktail of proteases (trypsin, chymotrypsin, and proteinase K) for various time points and the remaining infectivity of the samples was determined by infection of TZM-bl cells. The percent infectivity remaining was calculated relative to virus incubated without protease present for the same time periods.

In an attempt to reconstitute the phenotypes of the most stable variants, we combined the stabilizing mutations identified from the GB2 and HC11 viral pools to produce Gmut (I535M, L543Q, K574R and H625N/T626M) and Hmut (S649A and V1alt). We also generated comb-mut, which combines both sets of consensus mutations from the two unrelated stable clones. When challenged with GuHCl, both Gmut and Hmut clearly recapitulated the phenotype of the clones from which they were derived. Notably, comb-mut was even more stable than either variant, being resistant to GuHCl at 2 M (**Figure 12A**). Similar results were seen with heat treatment, although in this case comb-mut was only slightly more stable than the others. Similarly, following prolonged incubation at 37°C, all of the stabilized mutants showed much improved half-lives compared to wild-type ADA, but no significant differences could be seen between the mutants. Loss of infectivity due to heat or 37°C incubation involves multiple viral components and we have seen that functional Env stability beyond a T90 of ∼50°C or a half-life of ∼20 h at 37°C cannot be quantified under the conditions of the assay [7].

We further tested the functional stability of comb-mut Env using destabilizing ligands. ADA wild-type or comb-mut virus was pre-incubated with the same mAbs and inhibitors used in Figure 8 for two different time periods. The inhibition data was again plotted both as the ratio of IC50s after a one hour incubation to that of a 20 hour incubation, as well as the ratio of IC50s of comb-mut to that of wild-type ADA using just the one hour incubation time. Comb-mut resisted destabilization by sCD4, b12, 4E10, and 2F5, as seen previously with GB21-6, and likewise was more sensitive than wild-type ADA to PF-348089 (**Figure 12B and C**). However, comb-mut was even more resistant to inhibition by sCD4 than GB21-6, exhibiting an 80-fold increase in IC50 relative to wild-type virus. In order to verify that resistance to destabilizing mAbs is not due to changes in binding site accessibility or integrity, we measured binding of a panel of mAbs to virion-displayed Env in a simplified virus ELISA format [11]. From this panel, we found that all of the broadly neutralizing mAbs to gp120 bound at least somewhat more strongly to comb-mut virus than to ADA (**Table S2**). In particular, neutralizing mAbs that bind the outer surface of gp120 (*e.g.* PGT128, PG9, PG16, and 2G12) bound ∼10–50-fold more strongly to comb-mut than to ADA, while mAbs against the more recessed CD4bs bound ∼4-fold better. Binding of neutralizing mAbs to gp41 was equivalent between comb-mut and wild-type ADA. In contrast, all of the non-neutralizing mAbs tested in this assay exhibited somewhat reduced binding affinity to comb-mut virus relative to ADA. In particular, mAb 7B2 against the immunodominant disulfide loop region of gp41 showed 20-fold lower binding to comb-mut. These results show that functional comb-mut Env trimers are resistant to destabilization by various inhibitors and the binding sites of these inhibitors appear to be intact on virions, while the epitopes of non-neutralizing mAbs appear to be diminished on virions, although not eliminated.

**Figure 13:**
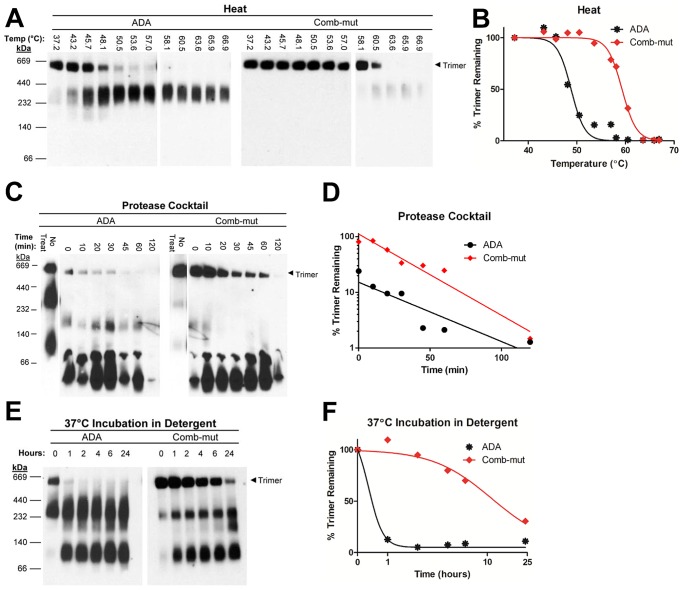
Comb-mut Env spikes exhibit increased oligomeric stability. (**A**) Wild-type ADA and comb-mut viruses were subjected to a heat gradient for 1 hour and then visualized using BN-PAGE. The Western blot shown was stained with an anti-gp41 cocktail of mAbs, and similar results were seen using anti-gp120 mAbs. (**B**) The intensity of the Env trimer bands from each lane in panel A was quantified using ImageJ software and plotted as the percent of the trimer band remaining relative to the corresponding band in the 37°C treated lane. The samples exposed to higher temperatures (58–67°C; the blot on the right for each virus) were run on a separate gel and the band intensities were calculated relative to 37°C treated controls on those gels that are not shown. (**C**) Wild-type ADA and comb-mut virions were incubated at 37°C with a protease cocktail as in **Figure 12D** and the oligomeric state of Env was analyzed by BN-PAGE. The Western blot was stained as in panel A. (**D**) Env trimer band intensities, as quantified for panel B. (**E**) Wild-type ADA and comb-mut virions were incubated at 37°C for up to 24 hours in 1% DDM detergent. The oligomeric state of DDM-treated Env was subsequently visualized using BN-PAGE. The Western blot was stained as in panel A. (**F**) Env trimer band intensities, as quantified for panel B.

We further examined the direct effect of heat on Env trimer dissociation by comparing wild-type ADA and comb-mut using BN-PAGE. As expected, the Env trimer of wild-type ADA dissociated on heat treatment at a temperature slightly above the T90 of ADA (**Figure 13A and B**) [7]. In contrast, the Env trimer of comb-mut was much more resistant to this treatment and did not significantly dissociate until an incubation temperature of 63.6°C. The observed increase in oligomeric stability of mutant Env trimers might be due at least in part to an increase in the level of uncleaved gp160 incorporated into the virus. We therefore analyzed the relative levels of cleaved gp120 and uncleaved gp160 associated with each virus by reducing SDS-PAGE. We observed no effect on cleavage as a result of the stabilizing mutations, as all Env variants appear to be ∼95% cleaved (**Figure S2**).

### Resistance to proteases is associated with increased stability of Env trimers

Immunization with virus particles typically does not elicit neutralizing Abs to the autologous virus, suggesting that native Env might be degraded rapidly *in vivo*. In addition to spontaneous dissociation, a possible cause of Env degradation is proteolysis. To explore their protease sensitivity, we treated ADA and comb-mut virions with a cocktail of trypsin, chymotrypsin, and proteinase K and then measured virus infectivity over a time course at physiological temperature. We note that with concentrated (500-fold) virus, the infectivities of ADA and comb-mut decreased much more rapidly than with unconcentrated virus, possibly due to an effect of lysosomal proteases or other cellular agents that might pellet with the virus. After normalizing for this effect, we found that ADA infectivity was reduced by ∼50% immediately upon treatment and the virus was almost completely inactivated after two hours (**Figure 12D**). In contrast, comb-mut was significantly more resistant to this treatment and viral infectivity did not drop below 60% of the untreated virus control over the same time period. When analyzed using BN-PAGE, the majority of protease-treated ADA Env trimer was already consumed at the earliest time point analyzed, while the effect on comb-mut Env trimers was significantly delayed and less complete (**Figure 13C and D**). Thus, the trimer-stabilizing mutations in comb-mut appear to make the Env complex less susceptible to degradation by a cocktail of different protease specificities.

### The stabilizing mutations slightly increase the proportion of secreted gp140 existing in a trimeric state

While membrane-anchored Env is arguably most relevant for structural studies and vaccine development, truncated gp140 trimers are also of considerable interest. However, the two forms are likely to have stability requirements that are at least somewhat different since the TM and viral membrane play critical roles in stabilizing native spikes. In addition, techniques employed to artificially trimerize gp140s have typically altered its conformation, which poses a conundrum as to which truncated forms of Env trimer to use to evaluate mutations that were selected in the membrane-anchored context. Rather than investigate artificial trimerization motifs, disulfide bonds, or cleavage site knockout mutations, we decided to determine the oligomerization state of secreted gp140s without further genetic modification. We chose to produce gp140s by transient transfection of 293S (GnTI−/−) cells that result in relatively homogeneous glycosylation (*i.e.* only Man5, Man8, and/or Man9), as ADA gp140 produced in GnTI−/− cells has been shown to form trimers, at least in the uncleaved form [49]. We generated cleavage-competent ADA and comb-mut Envs that were truncated after amino acid position 664, since trimers with this truncation have been shown to be relatively well-behaved [50]. We observed that ADA and comb-mut gp140 produced in 293S cells was indeed at least partially trimeric as measured by BN-PAGE, whereas the trimeric fraction was negligible when produced for comparison in 293T cells (**Figure S3A**). The Env constructs have an intact cleavage site between gp120 and gp41, so we wished to determine the actual level of cleavage in the soluble Env preparations using SDS-PAGE. We observed that both ADA and comb-mut soluble gp140s were approximately 50% processed (**Figure S3B**).

**Figure 14:**
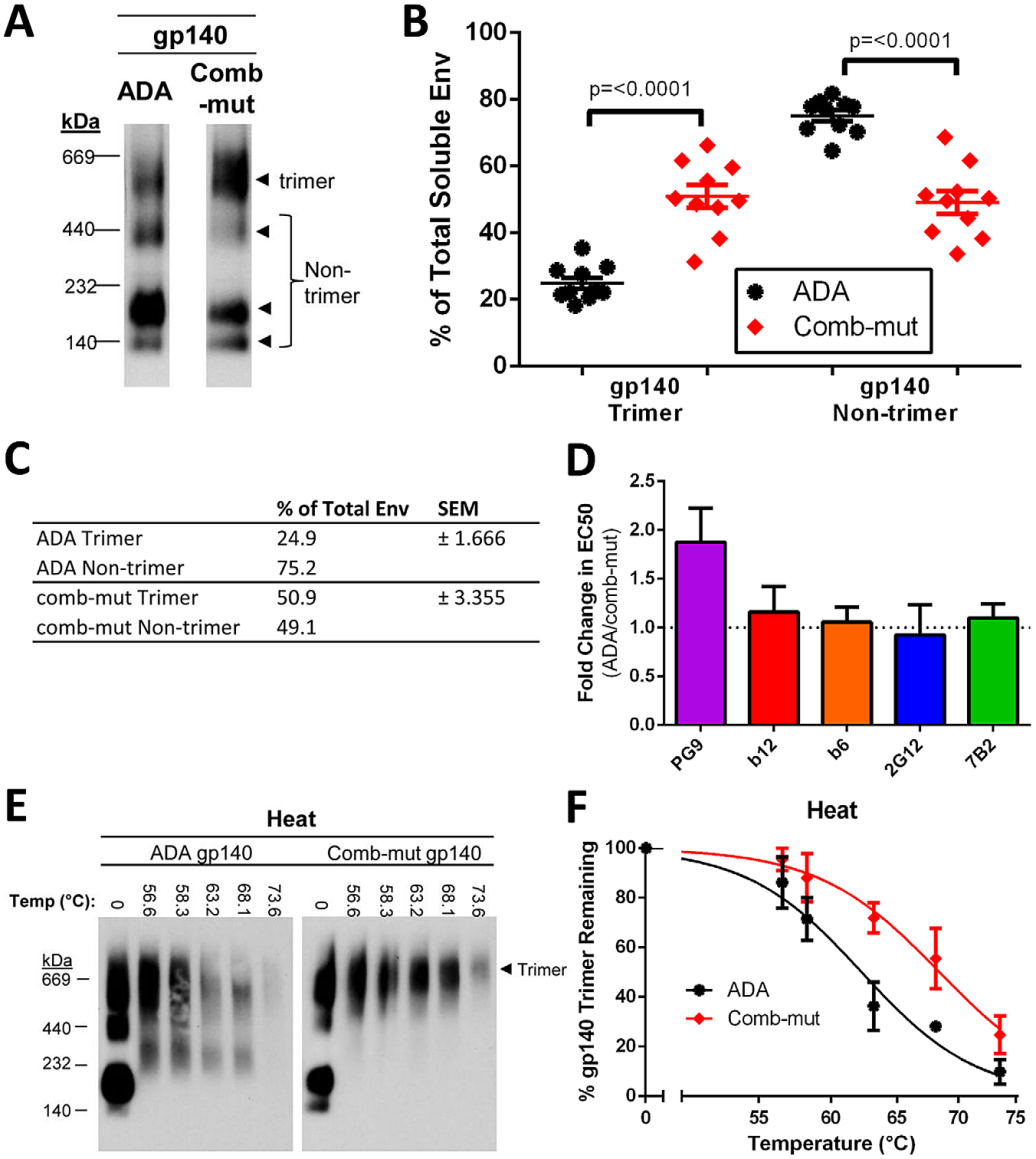
Comb-mut stabilizing mutations increase the proportion of trimers of gp140s secreted from 293S cells and increase mAb PG9 recognition. (**A**) The oligomeric state of ADA and comb-mut gp140 secreted from 293S (GnTI−/−) cells was analyzed by BN-PAGE using an anti-gp120 mAb cocktail. The gp140 trimer band was verified by size comparison to KNH1144 SOSIP and JRFL-foldon gp140 trimers. Other (non-trimeric) bands are postulated to correspond to gp140 dimer, gp140 monomer, and gp120 monomer, respectively, based on size, but this has not been confirmed. The blot shown is a representative example of ten separate BN-PAGE analyses of Env from three independent transfections. (**B**) The percentage of total density present in either the Env trimer band or in the non-trimeric Env bands combined was quantified for all BN-PAGE analyses and the differences between ADA and comb-mut were compared. (**C**) Data from panel B presented in numerical format. (**D**) Binding of a panel of mAbs to ADA wild-type and comb-mut gp140s from cell culture supernatant was determined using an ELISA. The fold change in binding EC50 to comb-mut relative to ADA (*i.e.* ADA EC50/comb-mut EC50) was calculated and plotted. For PG9, the difference in EC50 between ADA and comb-mut was statistically significant (p = 0.0012, n = 8). No significant difference in binding was found for the other mAbs. (**E**) ADA and comb-mut soluble gp140 samples were subjected to a heat gradient for 1 hour and then visualized using BN-PAGE. The experiment was performed three times and a representative Western blot is shown. (**F**) The intensity of the Env trimer bands from each lane in panel E was quantified and plotted as the percent of the trimer band remaining relative to the corresponding band of the sample incubated on ice. The results are the average of three independent experiments.

When the oligomeric states of secreted comb-mut and ADA gp140s were compared using BN-PAGE, we observed a statistically significant increase over wild-type in the proportion of comb-mut Env that spontaneously formed trimers (51% trimer for comb-mut and 25% trimer for ADA, p = <0.0001, n = 10), which was accompanied by a corresponding decrease in bands corresponding to non-trimeric Env (**Figure 14A, B, and C**). To rule out the possibility that this apparent increase in the trimeric population could be an artifact of BN-PAGE/Western blots, we used the same Env preparations in ELISA to analyze binding to PG9 - that has a strong preference for trimeric Env [41] - along with several control mAbs. With the control mAbs, we observed strong binding by both neutralizing (*e.g.* 2G12 and b12) and non-neutralizing (*e.g.* b6 and 7B2) control mAbs, with no change in binding between the two Envs with these or other mAbs (**Figure 14D** and data not shown). However, much weaker but highly reproducible binding was observed using PG9 against both ADA and comb-mut soluble Env, and, consistent with the BN-PAGE data, there was a statistically significant two-fold increase in PG9 binding to comb-mut relative to ADA (**Figure 14D**). Thus, the secreted Env is comprised of Env trimers that either lack certain antigenic features of native Env, or those Env trimers with native antigenicity would have to be a relatively minor constituent of the total Env population. However, in addition to slightly enhancing trimerization of soluble gp140, the stabilizing mutations in comb-mut also cause a small but significant increase in the proportion of PG9-reactive molecules, which are both sought after features in Env immunogen design.

To determine the stability of soluble trimers of comb-mut and wild-type gp140s, we assayed heat induced trimer dissociation using BN-PAGE (**Figure 14E**). Interestingly, the lower molecular weight species of Env disappeared from the Western blot at intermediate temperatures and appeared to form larger complexes, while the trimer band disappeared at higher temperatures. Corresponding monomeric dissociation byproducts did not appear concomitantly with the disappearance of the trimer band and all staining became undetectable after treatment at higher temperatures, presumably due to product aggregation. Under these conditions the comb-mut trimer did appear more stable than ADA, as it consistently disappeared from the blot at a higher temperature (i.e. 50% trimer disappearance at 68.4°C for comb-mut *vs.* 62.3°C for ADA; **Figure 14E and F**). Importantly, both ADA and comb-mut soluble gp140 trimers were much more thermostable than their functional Env trimer counterparts on the virion, as the gp140s retained a significant amount of trimer following incubation at 68°C for one hour.

Because the gp140s are largely unprocessed (**Figure S3B**), and because uncleaved Env has been shown to be more stable than its cleaved counterpart on the membrane surface [6], we wished to determine the stabilities of uncleaved and cleaved gp160s of ADA and comb-mut. We compared replication-competent virions that display mostly cleaved Env, pseudotyped virus particles that display mostly uncleaved gp160, and Env produced by DNA transfection in the absence of viral backbone that is essentially uncleaved (**Figure S4B and D**). When subjected to heat and visualized by BN-PAGE, the uncleaved gp160 formed a less discrete oligomeric band at 57°C, which appeared to increase in size at higher temperatures that cause cleaved ADA Env trimers to dissociate (**Figure S4A and C**). These results are quite similar to what was observed when soluble gp140s were exposed to heat, suggesting that uncleaved gp140 trimers may share some stability features with their uncleaved gp160 counterparts.

### Mutationally stabilized native Env spikes resist dissociation by detergent

Because the stability of native Env trimers is dependent on interactions with the membrane [7], we wished to investigate comb-mut Env stability in detergent. We previously showed that fully mature, virion-associated Env of a clade B primary isolate, JR-FL, dissociated at physiological temperature in under four hours following solubilization in the mild detergent, DDM [7]. We therefore used BN-PAGE to analyze the stability of ADA wild-type and comb-mut trimers in DDM over time at 37°C. ADA Env trimers quickly dissociated under these conditions and had almost completely decayed after one hour (**Figure 13E and F**). However, under identical conditions Env from comb-mut retained a substantial fraction of trimeric Env (30%) a full 24 hours following DDM treatment. Hence, the comb-mut Env spike is not only relatively resistant to heat, proteolysis and GuHCl treatment but also exhibits greatly increased stability after being detergent-solubilized.

## Discussion

HIV-1 has been experimentally subjected to various evolutionary selection pressures in order to study its fitness, tropisms, and various aspects of Env structure-function including mechanisms of escape from drugs and neutralizing antibodies [30], [33], [34], [51]. Here, we used directed evolution to identify amino acid changes in HIV-1 Env that increase the stability and homogeneity of the unliganded spike without grossly altering its function or its antigenic properties, and without the aid of structural data. Previous engineering approaches have sought to stabilize Env trimer-based immunogens using intermolecular disulfides, cleavage site knockouts, and artificial trimerization domains, but each approach has adversely affected the function and antigenic profile of the cognate native trimer [18], [28], [52], [53].

Selection strategies may be devised to identify HIV-1 Env trimers with even greater stabilities than we observed here. Env requires a degree of conformational flexibility in order to mediate fusion of the viral membrane with the target cell membrane [54]. Screening for stable Env trimers in the absence of an infectivity requirement may therefore identify a greater diversity of Env-stabilizing mutations. Nevertheless, such screens can also lead to non-native conformations of Env so specific counter-screens may also be necessary.

In our screen for Env stability, several trimer-stabilizing mutations were identified in the NHR of gp41. A prior study identified substitutions I535M and L543Q in the NHR that led to decreased levels of non-trimeric Env on pseudotyped virus, but native Env trimer stability was not explicitly measured [55]. In the current study we show that these substitutions in the NHR stabilize the functional form of HIV-1 Env. The K574R substitution we identified affects a highly conserved residue in the NHR, with >99.5% of group M isolates having a Lys at position 574 in the LANL HIV sequence database. Non-conservative substitutions with K574 tend to destabilize the post-fusion (6HB) conformation of gp41 [45], but their effect on unliganded trimer stability has not previously been studied. We show here that mutations to K574 can either stabilize (*e.g.* K574R) or destabilize (*e.g.* K574A) the unliganded, native trimer. Thus, position 574 appears to have a pivotal role in regulating multiple conformations of Env, which may explain the high degree of sequence conservation at this position. Which interactions this residue makes as it transitions between the unliganded and receptor primed forms of Env is unclear as the structural details of these states are currently lacking.

Post-CD4 engagement, the NHR region of gp41 forms a homotrimeric coiled-coil that is transiently accessible to peptide inhibitors and Abs [36], [56]. However, details of NHR structure in the unliganded (ground) state have not been described. The NHR appears to form a homo-trimer in receptor activated and post-fusion conformations of Env and has even been implicated in trimerization of the unliganded Env complex, though apparently not by equivalent mechanisms [55], [57]. The NHR mutations we identified here may enhance subunit-subunit interactions within Env, which could resist structural transitions out of the native state and into CD4-bound, antibody-bound, and other inactive states (see below). We speculate that while the K574R mutation maintains hydrophilicity and charge, the guanidino group may enhance electrostatic interactions or hydrogen bonds with adjacent elements on Env. The mutation L543Q, and to a lesser extent I535M, involve the substitution of a hydrophobic side-chain with a polar residue that is more likely to be found on the surface of the protein, suggesting that this portion of the NHR might be at least somewhat solvent-exposed and poised to interact with other hydrophilic elements. Cryo-electron microscopic (cryo-EM) structures of the Env trimer have shown the presence of a hole in the center of the trimer [58], [59], and a recent study suggests that the NHR helices may line this cavity [60].

We also identified Env stabilizing mutations in the CHR region of gp41: H625N, T626M and S649A. Residues N625 and M626 occur commonly among HIV-1 isolates. However, S649 is conserved in group M (95.3% of isolates), while A649 predominates in groups N and O and SIVcpz. A number of studies have implicated the CHR of gp41 in Env subunit-subunit interactions. Thus, mutations in the CHR can disrupt gp120-gp41 interactions and increase spontaneous shedding of gp120 [23]. In addition, a peptide corresponding to the DSL and CHR regions of gp41 can bind to monomeric gp120 through interactions with the C5 and C1 regions of gp120 [61]. Another study showed that peptides corresponding to gp120 C4 can interact with the peptide fusion inhibitor T-20, the latter of which is comprised mostly of CHR residues [62]. Residues in the CHR immediately N-terminal to position 646 have also been shown to contribute to gp41 trimerization [63]. Thus, the CHR in the unliganded, native trimer could conceivably interact with the inner domain and/or base of gp120 as well as with other gp41 protomers [58]. The presence of the dipeptide motif HT at positions 625/626 has been shown to increase virion infectivity in a CD4-independent manner and makes HIV-1 more sensitive to sCD4 and cold inactivation [64]. We note that, the substitution of NM for HT at positions 625/626 causes a putative N-glycosylation site (PNGS) to shift from N624 to the new N625. Studies have shown that this glycosylation site is occupied [50], [65], so its alteration might explain at least part of the stabilizing effect of this mutation. The S649A substitution involves a hydrophilic to hydrophobic residue change that could mean that this residue is in a more hydrophobic environment (*i.e.* buried) in the unliganded trimer.

The only stabilizing mutation identified in gp120 was the V1 alteration (N139/I140 deletion, N142S). In cryo-EM models, V1V2 is located at the apex of the trimer where it may interact with adjacent protomers by contacting other V1V2s [58], V3 [66] and/or other elements nearby on Env. V1 is heavily N-glycosylated and likely O-glycosylated as well [67]–[69]. The N142S mutation we identified eliminates a PNGS in V1, so glycosylation at this site and glycosylation proximal to this site may contribute to Env trimer stability as well.

The Env mutants that we identified in the B2 and HC11 library pools have distinct sequences and yet possess a stability phenotype that appears to be largely independent of the method of destabilization (*i.e.* GuHCl, heat, prolonged incubation at 37°C, destabilizing ligands, proteolysis, and detergent). Moreover, both Env mutants are hyper-sensitive to an entry inhibitor that opposes conformational changes in trimeric Env (*i.e.* PF-348089). Collectively, the results suggest that more than one element within Env may cooperate to resist trimer-destabilizing treatments, and that the different treatments may inactivate Env through a cooperative mechanism. We previously showed a correlation between heat stability and resistance of HIV-1 to 37°C decay, and, for the isolates JR-CSF and ADA, resistance to GuHCl also correlated with resistance to heat and spontaneous decay [7]. Recently, residues in gp41 including H625/T626 were found to increase CD4 independent infection, global neutralization sensitivity, and sensitivity of Env to cold inactivation [64]. Conformational changes in the Env trimer that lead either to infection or inactivation both involve an irreversible transition over an activation energy barrier [54], [70], [71]. The stability of native Env can either be increased by reducing the Gibbs free energy of the unliganded Env trimer or by increasing the free energy of the transition state that leads to a new state. It seems likely that the mutations we selected tighten interactions between subunits in the unliganded trimer. However, the mutations identified here may also destabilize the transition state that leads to inactive conformations, thus making it less likely that Env will decay. Elucidation of the mechanisms of Env stabilization might reveal structural distinctions between functional forms of HIV-1 Env trimers.

In addition to increasing the stability of Env, the mutations identified here also made the native Env trimer less sensitive to protease digestion. The mutations introduced into comb-mut are not expected to significantly impact the preferred cleavage sites of the enzymes tested. Most likely then the mutations in Env cause it to assume a more closed conformation in which protease cleavage sites are less accessible. It is possible that digestion of Env immunogens *in vivo* restricts elicitation of certain Abs [72], [73], so incorporation of comb-mut mutations into Env trimer-based immunogens might offer a level of protection against such degradation. We note that the specific enzymes used here would not be encountered at the site of vaccination, but comb-mut Env trimers are resistant to a cocktail of multiple proteases with different specificities so the effect may be more general. Binley and colleagues have shown that sequential glycosidase-protease digests can degrade non-functional Env species with greater efficiency than native Env trimers, but the process does reduce viral infectivity by ∼70% suggesting some effect on functional Env [11], [13]. By including stabilizing mutations in Env it may be possible to remove irrelevant Env without loss of native trimer.

As Env stability and Env homogeneity are not always correlated [6], [7], it is notable that the stable Env mutants selected in this study were also homogeneously trimeric on BN-PAGE. In support of the BN-PAGE results, the stabilized mutant Env virions were also captured with much lower efficiency than wild-type ADA using mAbs that bind poorly to unliganded native trimers (*i.e.* X5 and hNM01) [6], suggesting that less non-native Env exists on the stable ADA mutants. Reasons for the decrease in non-native Env may include less cellular biosynthesis of aberrantly folded or improperly glycosylated forms of Env prior to incorporation of Env onto the budding virion and/or slower decay of the folded Env trimer, both prior to budding from the cell and on the virus surface [7], [9], [13].

We found that mAbs PG9/16 neutralized the stable Env mutants of ADA more completely than the wild-type virus [10]. When certain PG9/16 resistant viruses are produced in GnTI−/− cells, PG9/16 can neutralize the resulting viruses more efficiently and completely, due at least in part to changes in glycans on the variable loops of gp120 that become enriched in Man5GlcNAc2 and oligomannose structures [10]. The stabilizing mutations therefore seem to reduce glycan heterogeneity within the functional population of Env. Mutations that stabilize the native trimer might increase the packing density of glycans and affect the ability of glycosylation enzymes to trim high mannose residues and add complex glycans [74], despite being distal from the actual glycosylation sites. It is notable that both GB21-6 and HC11-1 have completely different mutations, but both increase the proportion of PG9/16 sensitive Env.

Env spikes with high functional stability and high structural homogeneity might be useful for immunization studies [5]–[7], [13]. However, many factors besides trimer stability can influence immunogenicity of Env including mode and density of display, choice of adjuvant, ability to elicit T-cell help, and the capacity to stimulate the appropriate germline B cells and drive affinity maturation. Virus particles, while displaying native Env trimers, do so at low levels (∼10 copies/virion) and typically induce only weak neutralizing Ab titers [14], [75]. Env displayed at higher density (*e.g.* as soluble protein or on nanoparticles) may lower the affinity threshold of BCR activation by taking advantage of the avidity effect [76]. The Env-stabilizing mutations we identified increased both the trimerization and stability of secreted gp140s, although these effects were quite modest. However, we also show that this trimeric truncated form of Env is largely uncleaved and is more thermostable than that of native Env. Uncleaved Env trimers, whether membrane-anchored or soluble, can be relatively stable but have antigenic features that are not native-like (*i.e.* binding of non-neutralizing mAbs) [6]. Described soluble gp140 trimers that include artificial stabilizing alterations also differ antigenically from native Env [15]–[20], [49], [77]–[79]. One factor that might contribute to the non-native properties of secreted Env is that it may traffic in the cell differently from membrane-anchored protein, resulting in differences in folding, processing, and post-translational modification [74]. Other stabilizing modifications or re-routing of soluble gp140 through specific folding and processing pathways may be required to compensate for the gp41 TM/CT truncation.

As an alternative to membrane-anchored or soluble gp140s, detergent-solubilized Env spikes may be purified from virions and used in immunization or structural studies. A detergent solubilization strategy was recently used to prepare Env trimers for cryo-EM analysis, but uncleaved rather than cleaved Env trimers were used due to instability of the latter form of Env [80]. The mutations we identified here greatly increased the stability of fully cleaved virion spikes in detergent raising the possibility that cleaved Env trimers could also be purified. Future studies will be directed at how to incorporate native Env-stabilizing mutations into an immunogen that can elicit neutralizing Ab.

## Materials and Methods

### Reagents

#### (i) Plasmids

The molecular clone pLAI-ADA has been described previously [6]. It was generated by replacement of the *env* ectodomain (aa 24 to 692) of the pLAI.2 plasmid with that of the strain ADA (AAR05843 in Genbank). Env-complementation plasmids pcDNA-ADA and pcDNA-JR-FL were generated by subcloning *env* from pLAI-ADA and pLAI-JR-FL into the plasmid pcDNA3.1/V5-His-TOPO (Invitrogen). Plasmids containing RHPA4259 [81], Q769.b9 [82], and ZM109F [83] *env* were obtained from the National Institutes of Health AIDS Research and Reference Reagent Program (NIH ARRRP; contributed by B. Hahn, J. Overbaugh, and C. Derdeyn, respectively). Mutations to genes were introduced using Quikchange site-directed mutagenesis (Agilent) according to the manufacturer's directions.

#### (ii) Cells

TZM-bl cells were obtained from the NIH ARRRP (contributed by J. Kappes and X. Wu), and 293T cells were from the ATCC. Both cell lines were maintained in Dulbecco's Modified Eagle Medium (DMEM) containing 10% FCS, 20 mM L-glutamine, 100 U/ml penicillin and 100 µg/ml streptomycin. 293S cells, a GnTI−/− derivative of 293T cells [84], were obtained from I. Wilson (TSRI) and were maintained as above. These cells are deficient in *N*-acetylglucosaminyltransferase I and are unable to add complex clycans to Man_5_GlcNAc_2_, resulting in glycans that are mostly Man_5_. MT2-CCR5ΔCT cells were a kind gift from D. Mosier (TSRI) and were maintained as above using RPMI media instead of DMEM.

#### (iii) Antibodies and inhibitors

Anti-HIV-1 mAbs and inhibitors were obtained from the following sources (epitope/binding site specificities in parentheses): b12 (CD4 binding site; CD4bs) [85], b6 (CD4bs) [86], X5 (coreceptor binding site; CoRbs) [87], hNM01 (V3) [88], PGT128 (V3 and glycans) [89], PG9 and PG16 (V2 and V3) [41] IgGs were from D. Burton (TSRI); 2G12 (gp120 high-mannose glycans) [90], 4E10 (membrane proximal external region; MPER) [91], and 2F5 (MPER) [92] IgGs were from H. Katinger (Polymun); VRC01 (CD4bs) [93] IgG was from J. Mascola (Vaccine Research Center); F425 B4e8 (V3) [94] IgG was from L. Cavacini (Harvard Medical School); 7B2 (gp41 disulfide loop; DSL) [95], 19b (V3) [96] and 17b (CoRbs) [97] IgGs from J. Robinson (Tulane); D50 (C-heptad repeat; CHR) [98] IgG was obtained from P. Earl (NIH); 8K8 (N-heptad repeat; NHR) [48] and Z13e1 (MPER) [99] IgGs were produced in-house; T-20 (NHR) [100], C34 (NHR) [42], CD4-IgG2 (CD4bs) [101] and soluble CD4 (CD4bs) [102] were obtained from the NIH ARRRP, contributed by Roche, NIAID, and Progenics, respectively; PF-68742 (gp41 loop region) [44] and PF-348089 (CD4bs), an analogue of BMS-378806 [103], were from E. Murray (Pfizer).

### Virus production

Virus was produced from 293T cells by transient transfection using the polyethylene imine (PEI) as previously described [6]. When virus was amplified in MT2-CCR5ΔCT cells, cells were infected at an m.o.i. of 0.01. Every 2–3 days, one half of the cells and virus-containing media was removed and replaced with media containing fresh cells. This procedure was continued for 10–12 days.

### Generation of mutant HIV-1 Env ADA library pools

Oligonucleotide directed mutagenesis was used to generate HIV-1 ADA mutant pools by targeting four different regions of Env that have been shown to be involved in subunit-subunit interactions in Env. Mutagenesis was targeted to the C1 region of gp120 (pools C1 and C2), the β3–β5 loop of gp120 (pools B1 and B2), the disulfide loop region (DSL) of gp41 (pools D1, D2, and D3), and the membrane proximal external region (MPER) of gp41 (pool M) (**Figure S1A**). Mutagenesis was restricted to amino acid residues found to naturally occur in the Los Alamos National Laboratory (LANL) HIV Sequence Database. The libraries were created using two PCRs: one 3′ PCR using a primer containing degenerate codons in the region targeted and a 5′ PCR that would partially overlap with the 3′ PCR upstream of targeted region. These two PCR products were then joined by splicing-overlap-extension PCR and the mutant Envs were subcloned into the molecularly cloned HIV-1 Env display vector, pLAI-ADA. Randomly selected test clones were sequenced and each was found to be a unique variant containing between 1 and 8 mutations in the targeted region (**Figure S1B and C**). The bulk ligated DNA was used to transfect 293T cells and virus-containing cell culture supernatant was used to infect MT2-CCR5ΔCT cells at an m.o.i. of 0.01 to produce pools of replication-competent viruses.

### Selection of HIV-1 (ADA) for improved stability

Incremental concentrations of denaturant (0.25–2 M GuHCl or 0.5–4 M Urea), hyper-physiological temperatures (45.7–53.6°C), and incubation time periods at 37°C (4–6 days), designed to be in the range that inactivates wild-type ADA [7], were used separately to select the 8 virion pools in duplicate. In the case of denaturants, treated viruses were pelleted by centrifugation and the denaturant was washed away prior to the infection step. After the destabilizing treatment, an aliquot of each viral pool was analyzed for infectivity in TZM-bl cells to determine the proportion of virus inactivated, and the remaining virus was rescued on MT2-CCR5ΔCT cells. A total of three such rounds were performed for each virion pool.

### Rescue of HIV-1 Env clones using RT-PCR

Following 3 rounds of selection, individual clones were rescued from each stability-enhanced library pool. Whole RNA was isolated from virions in culture supernatant using the QIAamp Viral RNA kit (Qiagen). SMARTScribe Reverse Transcriptase (Clontech) was used to produce cDNA from the viral RNA using the primer NefOR (AGGCAAGCTTTATTGAGG; donated by D. Mosier, TSRI) which binds downstream from *env*. Next, *env* was amplified using the Expand High Fidelity PCR System (Roche) and Env-specific primers (*i.e.* pLAI5EnvF 5′-TAGGCATCTCCTATGGCAGGAAG-3′ and pLAI3EnvR 5′-GTCTCGAGATGCTGCTCCCACCC-3′). Amplified *env* was subcloned into pLAI-ADA using a *BamH* I and *Bgl* I restriction sites. Individual plasmid DNA, amplified in *E. coli*, was purified and full-length *env* was sequenced.

### Rescue of HIV-1 Env clones using limiting dilution

A serial 5-fold dilution was performed for each stability-enhanced virion pool and the virions were added to MT2-CCR5ΔCT cells. After 24 h, the media was replaced, and following a 7 day incubation, cell culture supernatants were harvested and tested for the presence of infectious virus in the TZM-bl assay. The media from the highest dilution to produce infectious virus was saved for stability tests.

### Stability treatment of HIV-1

Virions were exposed to incremental concentrations of GuHCl or urea for 1 h, increasing temperature for 1 h, or extended incubation at 37°C. Samples treated with denaturants were pelleted in a microcentrifuge (20,000×*g* at 4°C) and were washed with fresh media twice before being resuspended in an equal concentration of media. Virus was then added to TZM-bl cells and luciferase activity was determined 72 h later using the Bright-Glo System (Promega) and an Orion microplate luminometer (Berthold Instruments). Residual infectivity was determined, and results are expressed relative to untreated virus. All experiments were performed in triplicate.

### BN-PAGE Western blot

Virus used for BN-PAGE was pelleted in an Optima ultracentrifuge (Beckman; 60,000×*g* at 4°C) and resuspended 100-fold concentrated in PBS. Virions were exposed to destabilizing conditions as above. BN-PAGE was performed as previously described [7]. Briefly, samples were treated with 1% DDM for 20 min on ice, and then electrophoresed on 4–16% NativePAGE Bis-Tris gels (Invitrogen). Proteins in the gel were then transferred to a PVDF membrane, membranes were blocked in 5% non-fat dry milk and blotted overnight at 4°C using a cocktail of mAbs to gp120 (b12, 2G12 and F425-B4e8, 2 µg/ml) or to gp41 (2F5, 4E10 and Z13e1 each at 1 µg/ml). After washing, membranes were probed for 30 min at room temperature with a goat anti-humanFc-HRP conjugated Ab (Jackson), and peroxidase activity was assayed using Super Signal West Pico Chemiluminescence (Pierce).

### Protease treatment

HIV-1 virions were concentrated 500-fold. The following proteases were added in Trypsin buffer (50 mM Tris-HCl, 20 mM CaCl2, pH 8.0): trypsin (50 µg/ml), chymotrypsin (50 µg/ml), and proteinase K (1 mg/ml; all NEB). Virions were incubated at 37°C for the indicated time periods and the digestion was stopped by addition of Complete Protease Inhibitor Cocktail (Roche) and stored at −80°C until analyzed. Samples were analyzed for infectivity and by BN-PAGE.

### Env trimer decay in detergent

Env was solubilized from virus particles by addition of n-Dodecyl β-D-maltoside (DDM) to a final concentration of 1% at 37°C. Samples were removed at different time points and analyzed using BN-PAGE as described above.

### Virus capture assay (VCA)

VCAs were modified from a previously detailed protocol [6]. Microtiter wells were coated overnight at 4°C with capture mAb (5 µg/ml in 50 µl of PBS). Wells were blocked using 4% non-fat dry milk (NFDM) in PBS for 1 h at 37°C. Incremental concentrations of soluble CD4 were added to 50 µl of virus in cell culture supernatant and, after a 15 min incubation, virions were added to the blocked wells and incubated for 2 h at 37°C. Wells were washed 6 times with PBS, and TZM-bl target cells were overlaid (10^4^ cells/well). Luciferase activity was determined after a 72 h incubation as described above.

### HIV-1 neutralization assay

HIV-1 infectivity and neutralization was determined as described previously [6]. Briefly, TZM-bl reporter cells were seeded in 96-well plates at 10^4^ cells per well in 100 µl complete DMEM and incubated for 24 h at 37°C. Virus samples were incubated with mAbs or inhibitors for 1 h or 20 h at 37°C, and the mixture was added to cells in a total volume per well of 200 µl. Cells were harvested 72 h post-infection, luciferase activity in the cells was determined as above.

### Virus ELISA

The virus ELISA was adapted from a previously described protocol [11]. Virions were immobilized directly on microtiter wells for 2 hours at 37°C (2 ng p24 equivalents per well). Plates were washed (all washes were performed using PBS without detergent) and wells were blocked using 4% NFDM in PBS for 1 h at 37°C. After washing, primary Abs were added in PBS containing 0.4% NFDM for 1 h at 37°C. Plates were washed again, goat anti-human-Fcγ-HRP secondary Ab (Jackson) was added, and the plates incubated for 45 min at 37°C. Following another wash, TMB substrate (Pierce) was added and absorbance read at 450 nm.

### gp140 production

ADA wild-type and comb-mut gp140 Env expression vectors were generated by introducing mutations D664G and K665stop in pcDNA-ADA using Quikchange site-directed mutagenesis (Agilent). Cleavage-competent gp140 proteins were produced by transient transfection of 293T and 293S (GnTI−/−) cells as described above for virus production. The oligomeric state of soluble gp140 in cell culture supernatant was analyzed by BN-PAGE and Western blot using only the anti-gp120 mAb cocktail, because the anti-gp41 mAbs used for Western blot staining bind to the region of gp41 removed by the truncation after position 664. The identity of the Env trimer band was verified by comparison with KNH1144 SOSIP [104] and JRFL-foldon soluble trimers [17] (gifts from I. Wilson and R. Wyatt (TSRI), respectively). Relative density of the BN-PAGE bands was analyzed using ImageJ software (NIH) and compared by t-test using GraphPad Prism.

### Gp140 ELISA

Microtiter wells were coated with *Galanthus nivalis* lectin (GNL; Sigma) at 5 µg/ml in PBS overnight at 4°C. Plates were then washed using PBS containing 0.05% Tween (PBST); all washes are with PBST. Plates were blocked with 4% non-fat dry milk (NFDM) in PBS for 1 h at 37°C. Next, plates were washed and gp140 cell culture supernatant was added for 2 h at 37°C. Following this incubation, plates were washed and mAb binding was assayed as with the Virus ELISA above, except that 0.05% Tween was included in all steps.

## Supporting Information

Figure S1 **Mutagenesis libraries created in HIV-1 Env ADA.** (**A**) A diagram of the major regions of Env and the locations targeted by partially degenerate mutagenesis primers (not shown) that were used on ADA Env to create libraries C1, C2, B1, B2, D1–D3 and M. The numbers below each library name are the exact amino acids targeted (HxB2 numbering). (**B**) Table showing the number of randomly selected clones that were characterized for each library and whether these were infectious in a single-cycle infectivity assay using TZM-bl cells. (**C**) Randomly selected test clones from the D2 library were sequenced in the mutagenic region. The ADA wild-type sequence is shown at the top and the amino acid changes from wild-type sequence in the test clones are highlighted in orange. The infectivity of the test clones relative to ADA wild-type is also shown. Similar results were observed with the other libraries. (**D**) Infectivities of the virion libraries produced by transfection of 293T cells and after passaging in MT-2 cells relative to an equivalent amount (p24) of wild-type ADA.
(TIF)

Figure S2 **Stable ADA Env variants show equivalent levels of processing as wild-type Env.** The cleavage of Env (gp160) from ADA, HC11-1, GB21-6, and comb-mut virions (all produced by transfection using the molecular clone plasmid pLAI) was analyzed by reducing SDS-PAGE followed by Western blot using an anti-gp120 mAb cocktail. The cleaved gp120 and uncleaved gp160 bands are indicated. The percent cleavage was quantified by measuring the relative intensity of the two bands using ImageJ software.
(TIF)

Figure S3 **Gp140 produced in 293S cells spontaneously forms a greater proportion of trimers when compared with 293T cells.** (**A**) The oligomeric state of ADA and comb-mut gp140 secreted by both 293S (GnTI−/−, left) and 293T cells (right) was analyzed by BN-PAGE using an anti-gp120 mAb cocktail. The bands were identified and labeled as in **Figure 14**. (**B**) The level of cleavage for ADA and comb-mut gp140s produced in 293S cells was determined using reducing SDS-PAGE as in **Figure S2**.
(TIF)

Figure S4 **Membrane-incorporated uncleaved gp160 oligomers are more thermostable than native Env trimers.** ADA (**A** and **B**) and Comb-mut (**C** and **D**) Env was expressed on replication competent, molecularly cloned virus (MC), pseudotyped virus (PSV), or by Env-complementation plasmid alone. All preparations were pelleted under the same conditions used to concentrate virus, which, in the case of the “Env only” sample, only concentrates microvesicles that are similar in size to HIV-1 and also associate with some forms of Env. Samples were then analyzed by BN-PAGE and SDS-PAGE Western blots using the same anti-gp120 mAb cocktail that was used in **Figure 14**.
(TIF)

Table S1 **Inhibition of stable HIV-1 Env mutants GB21-6 and HC11-1 by a panel of neutralizing mAbs and inhibitors.**
(DOCX)

Table S2 **The binding of a panel of mAbs to immobilized comb-mut and ADA virions assessed using virus ELISA.**
(DOCX)
