# Supplementary figures and images for: Increased Functional Stability and Homogeneity of Viral Envelope Spikes through Directed Evolution
Source: PLoS Pathog. 2013 Feb 28;9(2):e1003184. doi: 10.1371/journal.ppat.1003184 (PMC3585149; doi:10.1371/journal.ppat.1003184)

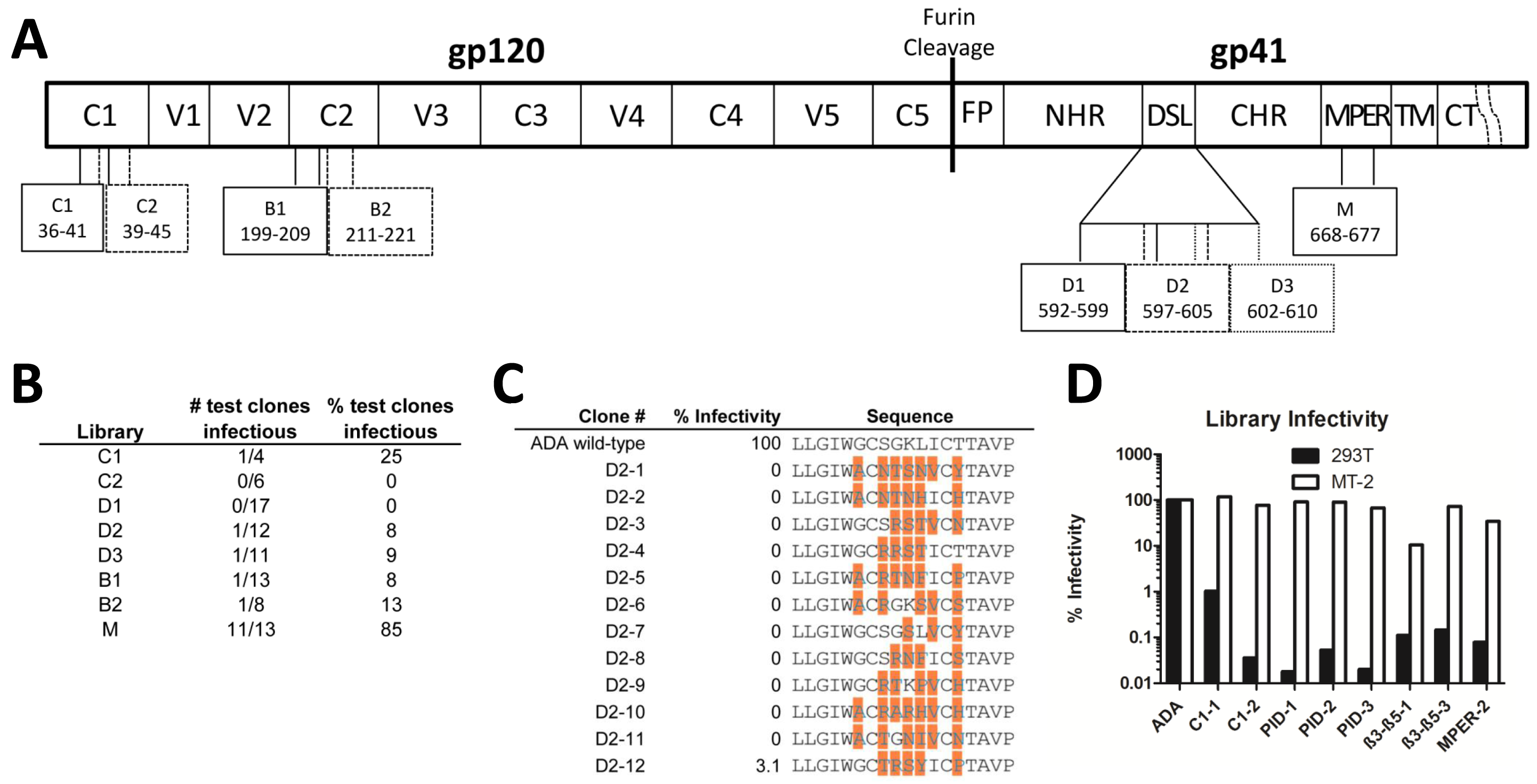

Supplement: Figure S1 — Mutagenesis libraries created in HIV-1 Env ADA. (A) A diagram of the major regions of Env and the locations targeted by partially degenerate mutagenesis primers (not shown) that were used on ADA Env to create libraries C1, C2, B1, B2, D1–D3 and M. The numbers below each library name are the exact amino acids targeted (HxB2 numbering). (B) Table showing the number of randomly selected clones that were characterized for each library and whether these were infectious in a single-cycle infectivity assay using TZM-bl cells. (C) Randomly selected test clones from the D2 library were sequenced in the mutagenic region. The ADA wild-type sequence is shown at the top and the amino acid changes from wild-type sequence in the test clones are highlighted in orange. The infectivity of the test clones relative to ADA wild-type is also shown. Similar results were observed with the other libraries. (D) Infectivities of the virion libraries produced by transfection of 293T cells and after passaging in MT-2 cells relative to an equivalent amount (p24) of wild-type ADA. (TIF) [file ppat.1003184.s001.tif]

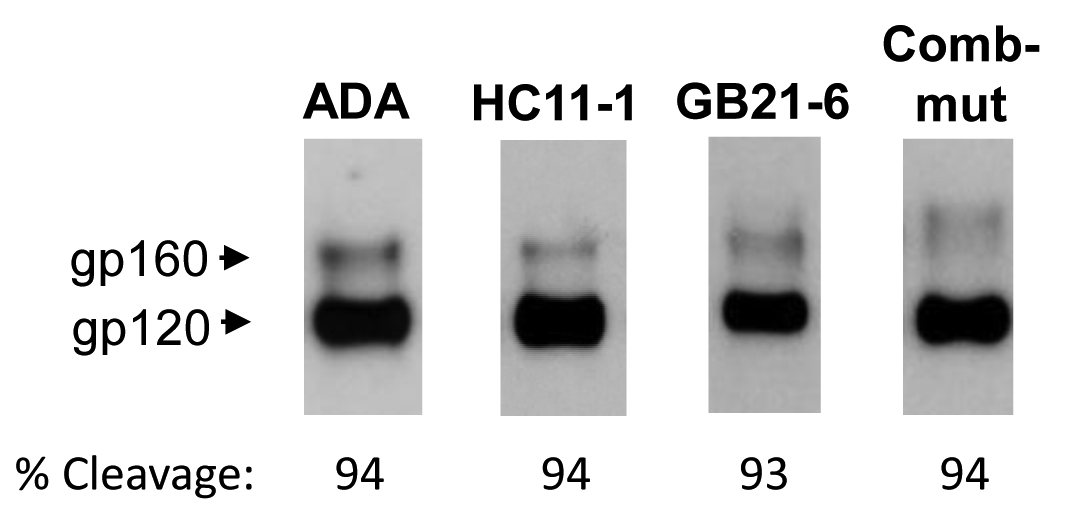

Supplement: Figure S2 — Stable ADA Env variants show equivalent levels of processing as wild-type Env. The cleavage of Env (gp160) from ADA, HC11-1, GB21-6, and comb-mut virions (all produced by transfection using the molecular clone plasmid pLAI) was analyzed by reducing SDS-PAGE followed by Western blot using an anti-gp120 mAb cocktail. The cleaved gp120 and uncleaved gp160 bands are indicated. The percent cleavage was quantified by measuring the relative intensity of the two bands using ImageJ software. (TIF) [file ppat.1003184.s002.tif]

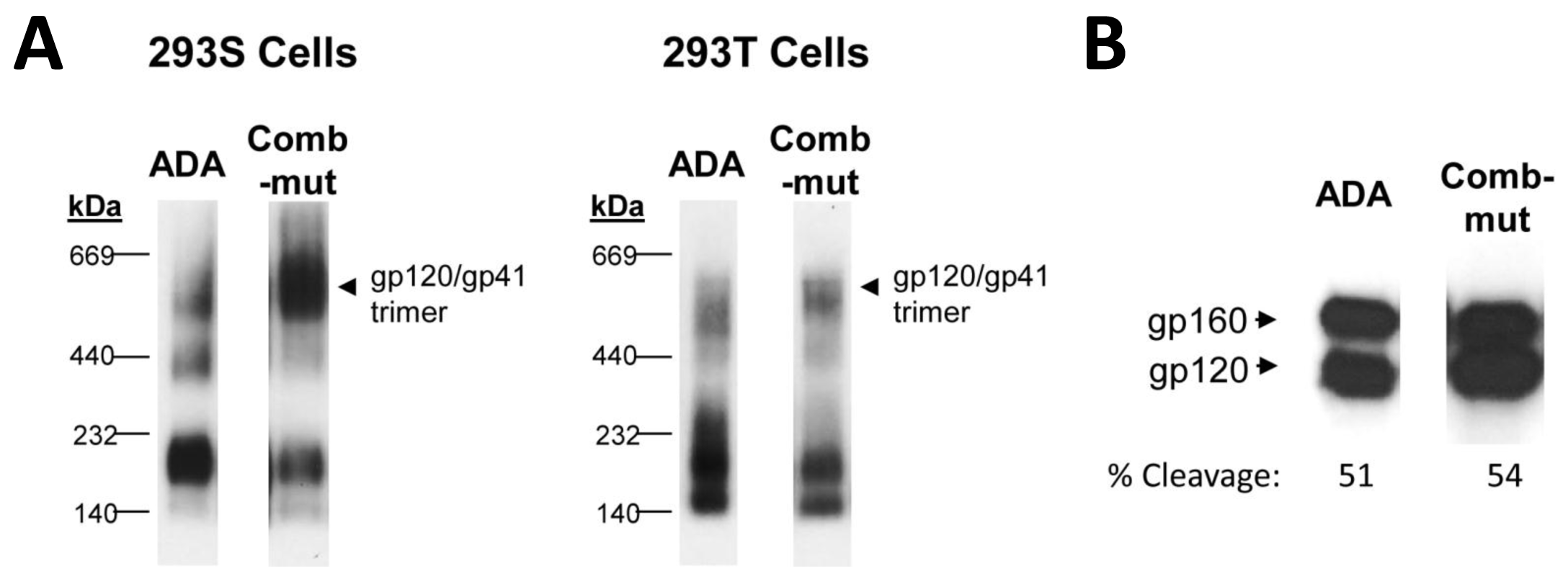

Supplement: Figure S3 — Gp140 produced in 293S cells spontaneously forms a greater proportion of trimers when compared with 293T cells. (A) The oligomeric state of ADA and comb-mut gp140 secreted by both 293S (GnTI−/−, left) and 293T cells (right) was analyzed by BN-PAGE using an anti-gp120 mAb cocktail. The bands were identified and labeled as in Figure 14 . (B) The level of cleavage for ADA and comb-mut gp140s produced in 293S cells was determined using reducing SDS-PAGE as in Figure S2. (TIF) [file ppat.1003184.s003.tif]

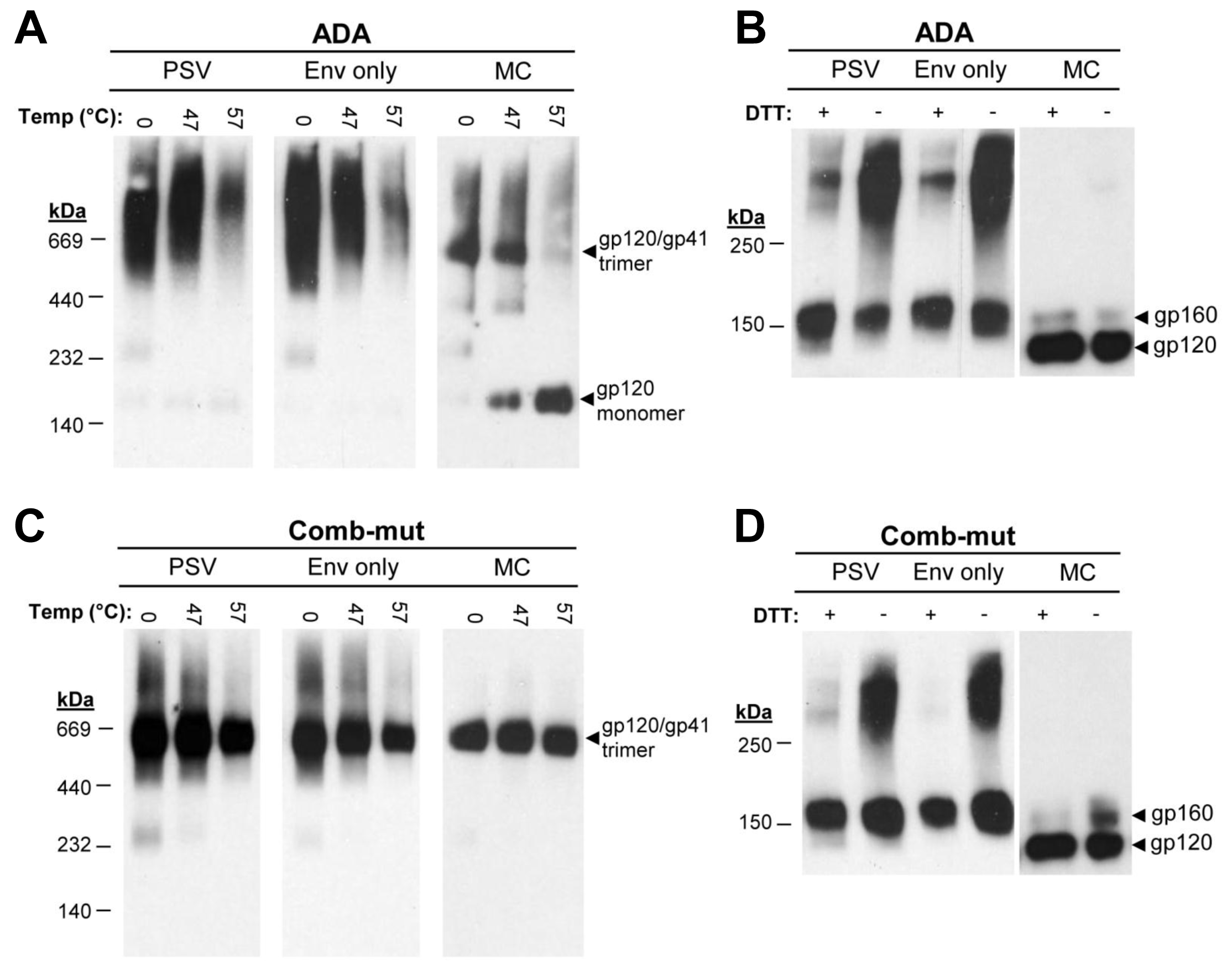

Supplement: Figure S4 — Membrane-incorporated uncleaved gp160 oligomers are more thermostable than native Env trimers. ADA (A and B) and Comb-mut (C and D) Env was expressed on replication competent, molecularly cloned virus (MC), pseudotyped virus (PSV), or by Env-complementation plasmid alone. All preparations were pelleted under the same conditions used to concentrate virus, which, in the case of the “Env only” sample, only concentrates microvesicles that are similar in size to HIV-1 and also associate with some forms of Env. Samples were then analyzed by BN-PAGE and SDS-PAGE Western blots using the same anti-gp120 mAb cocktail that was used in Figure 14 . (TIF) [file ppat.1003184.s004.tif]
