# Supplementary material for: Increased Functional Stability and Homogeneity of Viral Envelope Spikes through Directed Evolution
Source: PLoS Pathog. 2013 Feb 28;9(2):e1003184. doi: 10.1371/journal.ppat.1003184 (PMC3585149; doi:10.1371/journal.ppat.1003184)
Supplement: Table S1 — Inhibition of stable HIV-1 Env mutants GB21-6 and HC11-1 by a panel of neutralizing mAbs and inhibitors. (DOCX) [file ppat.1003184.s005.docx]

**Supplementary Table S1.** Inhibition of stable HIV-1 Env mutants GB21-6 and HC11-1 by a panel of neutralizing mAbs and inhibitors.

|  | **IC50 (nM)** | | | | | |
| --- | --- | --- | --- | --- | --- | --- |
|  | **ADA** | | **GB21-6** | | **HC11-1** | |
| **Inhibitor** | **1 hour** | **20 hour** | **1 hour** | **20 hour** | **1 hour** | **20 hour** |
| sCD4 | 17 | 1.2 | 500 | 170 | 66 | 11 |
| b12 | 0.55 | 0.037 | 10 | 1.6 | 1.5 | 0.19 |
| 4E10 | 3.5 | 0.39 | 42 | 11 | 4.1 | 1.1 |
| 2F5 | 1.6 | 0.13 | 23 | 4.2 | 3.6 | 0.78 |
| VRC01 | 1.8 | 0.35 | 1.6 | 0.22 | 1.5 | 0.33 |
| PG9 | 1.4 | 0.15 | 1.0 | 0.14 | 0.35 | 0.11 |
| C34 | 14 | 13 | 30 | 27 | 9.5 | 9.8 |
| PF-348089 | 120 | 120 | 13 | 10 | 20 | 26 |
