# Supplementary material for: Increased Functional Stability and Homogeneity of Viral Envelope Spikes through Directed Evolution
Source: PLoS Pathog. 2013 Feb 28;9(2):e1003184. doi: 10.1371/journal.ppat.1003184 (PMC3585149; doi:10.1371/journal.ppat.1003184)
Supplement: Table S2 — The binding of a panel of mAbs to immobilized comb-mut and ADA virions assessed using virus ELISA. (DOCX) [file ppat.1003184.s006.docx]

**Supplementary Table S2.** The binding of a panel of mAbs to immobilized comb-mut and ADA virions assessed using virus ELISA.

|  | **Neutralizing mAbs*** | | | | **Non-neutralizing mAbs** | |
| --- | --- | --- | --- | --- | --- | --- |
|  | **mAb** | | **EC50 Fold Change (ADA:Comb-mut)^†^** | | **mAb** | **EC50 Fold Change (ADA:Comb-mut)** |
| anti-gp120 | PGT128 | 11 | | b6 | | 0.26 |
|  | PG9 | 21 | | 19b | | 0.61 |
|  | PG16 | 48 | | 17b | | 0.28 |
|  | 2G12 | 20 | |  | |  |
|  | VRC01 | 3.1 | |  | |  |
|  | b12 | 4.2 | |  | |  |
|  | CD4-IgG2 | 4.4 | |  | |  |
| anti-gp41 | 4E10 | 0.73 | | 7B2 | | 0.05 |
|  | 2F5 | 1.2 | | D50 | | 0.80 |

* Neutralizing: IC50 < 50 µg/ml, Non-neutralizing: IC50 > 50 µg/ml.

† Fold change in mAb EC50 against comb-mut relative to ADA wild-type. Fold change greater than 1 signifies an increase in binding, while a fold change less than 1 represents a decrease in binding.
